# The activation of the piriform cortex to lateral septum pathway during chronic social defeat stress is crucial for the induction of behavioral disturbance in mice

**DOI:** 10.1038/s41386-024-02034-7

**Published:** 2024-12-05

**Authors:** Yuki Okuda, Dongrui Li, Yuzuki Maruyama, Hirokazu Sonobe, Tomoyuki Mano, Kazuki Tainaka, Ryota Shinohara, Tomoyuki Furuyashiki

**Affiliations:** 1 Division of Pharmacology, Kobe University Graduate School of Medicine, Kobe, 650-0017 Japan; 2 Computational Neuroethology Unit, Okinawa Institute of Science and Technology (OIST) Graduate University, Okinawa, 904-0412 Japan; 3 Department of System Pathology for Neurological Disorders, Brain Research Institute, Niigata University, Niigata, 951-8585 Japan

**Keywords:** Stress and resilience, Depression

## Abstract

Chronic stress induces neural dysfunctions and risks mental illnesses. Clinical and preclinical studies have established the roles of brain regions underlying emotional and cognitive functions in stress and depression. However, neural pathways to perceive sensory stimuli as stress to cause behavioral disturbance remain unknown. Using whole-brain imaging of Arc-dVenus neuronal response reporter mice and machine learning analysis, here we unbiasedly demonstrated different patterns of contribution of widely distributed brain regions to neural responses to acute and chronic social defeat stress (SDS). Among these brain regions, multiple sensory cortices, especially the piriform (olfactory) cortex, primarily contributed to classifying neural responses to chronic SDS. Indeed, SDS-induced activation of the piriform cortex was augmented with repetition of SDS, accompanied by impaired odor discrimination. Axonal tracing and chemogenetic manipulation showed that excitatory neurons in the piriform cortex directly project to the lateral septum and activate it in response to chronic SDS, thereby inducing behavioral disturbance. These results pave the way for identifying a spatially defined sequence of neural consequences of stress and the roles of sensory pathways in perceiving chronic stress in mental illness pathology.

## Introduction

Environmental factors, such as trauma and stressful life events, contribute to the risk of onset, progression, and relapse of mental illnesses, including depression [1, 2]. Rodent studies with chronic stress models, such as chronic unpredictable stress and chronic social defeat stress (SDS), have identified neural-circuit mechanisms underlying depression-related behaviors [3–5]. These studies have revealed various effects of chronic stress in multiple brain areas. Thus, it is widely accepted that chronic stress induces dendritic atrophy and decreases dendritic spines and excitability in the hippocampus and the medial prefrontal cortex (mPFC), whereas it causes opposite effects in the nucleus accumbens and the amygdala [6–9]. These structural and functional alterations are thought to underlie emotional and cognitive disturbance. These findings in rodents align with clinical observations that volumes and activities of the corresponding brain regions are altered in depressive patients [10–13].

Notably, these effects of stress vary depending on its duration and intensity. In rodents, acute stress reportedly increases dendritic branches and spines in mPFC pyramidal neurons via the dopamine D1 receptor to suppress behavioral disturbance [14], whereas chronic stress induces dendritic atrophy and spine loss through neuroimmune mechanisms, leading to behavioral disturbance [15]. This notion underscores the importance of elucidating neural responses to chronic, rather than acute, stress to understand mental illness pathology.

Since sensory information of external stimuli needs to be first received and processed in sensory regions [16], abnormalities in these regions may underlie emotional and cognitive disturbance in mental illnesses. Indeed, depressive patients often experience impairments in olfactory, auditory, and visual processing [17–22]. Music and bright light therapies have been used to help manage and treat depression [23–25]. Aromatherapy can also be used to effectively reduce depressive symptoms [26]. A few rodent studies recently reported the effects of stress on olfactory, visual, and auditory cortices and their behavioral relevance [27–31]. However, none of these studies have explored how animals perceive external stimuli as stress to induce behavioral disturbance.

To unbiasedly profile neural responses to acute and chronic SDS, we performed three-dimensional whole brain imaging of Arc-dVenus neural response reporter mice with tissue clearing and automated registration to the standard brain atlas. Our findings revealed that widely distributed brain regions differently contribute to neural responses to acute and chronic SDS. Among these regions, sensory cortices, especially the piriform cortex, primarily contributes to classifying neural responses to chronic SDS. Chemogenetic manipulation of neuronal activities show that the activation of the piriform cortex to lateral septum pathway underlies the perception of stress-associated olfactory stimuli to induce behavioral disturbance.

## Materials and Methods

### Animals

Male C57BL/6 N mice (7–8 weeks old) and retired male ICR breeding mice were purchased from Japan SLC (Shizuoka, Japan) and maintained in our animal vivarium for at least one week before experiments. Arc-dVenus mice [32] were obtained from Dr. Shun Yamaguchi at Gifu University, Gifu, Japan, and maintained in our breeding colony. Arc-dVenus mice were back-crossed to C57BL/6 N mice more than six times and maintained by breeding with C57BL/6 N mice. C57BL/6 N and Arc-dVenus mice were group-housed (three mice per cage) under a 12 h light/dark cycle with ad libitum access to food and water. All animal care and experimental procedures were conducted in accordance with the National Institutes of Health Guide for the Care and Use of Laboratory Animals and approved by the Animal Experimentation Committee of Kobe University.

### Social defeat stress (SDS)

SDS was performed as previously described [33] with minor modifications. Male ICR mice were screened based on their aggressiveness to a male C57BL/6 N mouse, as measured by attack latency and number in 3 min daily sessions over three days. Only those that showed an appropriate level of aggressiveness were used as aggressor. Experimental subjects, either male C57BL/6 N or Arc-dVenus mice, group housed in threes, were subjected to acute and chronic SDS by introducing a selected ICR mouse into their home cages. For acute SDS, SDS was applied for one hour only once. For chronic SDS, SDS was applied for one hour daily for 6 consecutive days. Different ICR mice were used on consecutive days. The ICR mouse was removed at the completion of SDS, and the experimental mice were left undisturbed until the next SDS or further examinations. The ICR mouse was replaced by a new one during SDS, if the aggression was either absent or excessive. Naïve mice as control were left undisturbed in their home cages until experiments.

### Social interaction test

Social interaction test was performed as previously described [14]. To habituate to a test environment, an experimental mouse was put into an open rectangular chamber (30 cm × 40 cm) containing an empty metal mesh cage (10 cm × 6 cm) at one end under a 10-lux red light illumination, and locomotor activity was measured for 150 s. To measure the level of social interaction, an experimental mouse was returned into the same chamber for 150 s, but a novel male ICR mouse was enclosed within the metal mesh cage. The trajectory of mouse ambulation was video-recorded and analyzed using SMART video tracking software (PanLab Harvard Apparatus). A rectangular zone of 30 cm × 15 cm containing the metal mesh cage was defined as the social interaction zone, and the 30 cm × 9 cm rectangular zone opposite to the social interaction zone was defined as the social avoidance zone. After the habituation or test period, mice were kept temporarily in a waiting cage until returning to their home cage when all mice from the same cage completed the test. This procedure consistently detected chronic SDS-induced social avoidance across all experiments, except for the one presented in Fig. 5. Although the cause of this inconsistency remains unclear, a key difference was noted in the surgical procedure: only in the experiment shown in Fig. 5 did the needle insertion for viral injection into the lateral septum cause severe damage to the superior sagittal sinus, leading to significant bleeding and the death of several mice within 24 hours post-surgery. This surgical complication may have acted as a significant stressor, potentially affecting the behavioral outcomes of chronic SDS.

### Female urine sniffing test

Female urine sniffing test was performed as previously described [34]. After individually housed for 30 min in the experimental room (illuminated with a 3-lux white light), mice were habituated to a water-soaked cotton-tipped applicator for 60 min. To measure the preference to female urine over water, the mice were exposed to a fresh water-soaked applicator for 5 min, and after a 45-min interval, to an applicator soaked with fresh female urine for 5 min. Female urine was taken from adult virgin female C57BL/6 N mice during the test period. Each session was video-recorded, and the time spent sniffing the cotton-tipped applicator soaked with water or female urine was measured.

### Novel object recognition test

Novel object recognition test was performed as previously described [35]. After one hour in the behavioral testing room (illuminated with a 15-lux white light), a mouse was kept in an open field chamber (30 cm × 40 cm) containing two identical Lego blocks. Following a four-hour interval, the mouse was returned into the same chamber with one of the Lego blocks replaced by a sand-filled flask. After each session, mice were kept temporarily in a waiting cage until returning to their home cage when all mice from the same cage completed the test. In each session, mouse behavior was video recorded for 10 min or until the mouse sniffed the objects for 20 s in total for post-hoc analysis. In the first session, the percentage of the time spent sniffing the right Lego block was calculated as the right object preference score. In the second session, the percentage of the time spent sniffing the sand-filled flask was calculated as the novel object preference score.

### Odor discrimination test

Odor discrimination test was performed as previously described [36]. After individually habituated to dry cotton swabs inserted into clean cages for 45 min, mice were presented with two odorants, almond extract and banana extract, in sequence. Each odorant of 50 µl diluted at 1:100 in water was applied to a fresh cotton swab and presented for 2 min. This odor presentation was repeated three times with 1-min intervals. The time spent sniffing an odorant was measured during each session for 2 min. After the test, mice were kept temporarily in a waiting cage until returning to their home cage when all mice from the same cage completed the test.

### Whole brain imaging and its computational analysis

Tissue clearing, whole brain imaging, and its computational analysis were performed as previously described [37–41]. Details are described in Supplementary Materials and Methods.

### Immunofluorescent staining

Immunofluorescent staining was performed as previously described [42] with minor modifications. Details are described in Supplementary Materials and Methods.

### Stereotaxic surgery

Stereotaxic surgery was performed as previously described [14]. After mice were anesthetized with 2.5–3.5% isoflurane (095-06573, FUJIFILM Wako Pure Chemical Corporation). The fur over the skull was removed, and the eyes were coated with ophthalmic ointment. The skull was then fixed with a stereotaxic chamber (Stoelting). During the surgery, the mice were maintained under anesthesia with 1–1.5% isoflurane, and their body temperature was regulated to 37 ^o^C using a heat pad. After the skin over the skull was sterilized, craniotomies were made using a drill (Muromachi Kikai) with a 0.8 mm bit. A glass micropipette made by a PN-30 micropipette puller (Narishige), filled with viral solution or cholera toxin subunit B (CTB) solution, was slowly lowered 500 μm past a target brain coordinate and returned to it. Coordinates are in mm relative to the bregma for anteroposterior (AP) and mediolateral (ML) or skull surface at the bregma for dorsoventral (DV), based on the Paxinos and Franklin mouse brain atlas[39]. Coordinates for the anterior piriform cortex (AP: +2.0, ML: ± 2.0, DV: −4.25), the posterior piriform cortex (AP: -1.7, ML: ±3.6, DV: −5.0), and the lateral septum (AP: +0.75, ML: ± 0.3, DV: −3.3), and the non-specific thalamic nuclei (AP: −1.05, ML: ± 0.00, DV: −3.75) were used. Then, the solution was pressure-injected at 100 nl/min using a PV-830 Pneumatic PicoPump (World Precision Instruments). After 5 min for the solution to diffuse, the glass micropipette was slowly removed, and the incision was sutured with a suture (Kono Seisakusho). After recovered from anesthesia, the mice were kept in group until experiments.

### Retrograde neuronal labeling with cholera toxin subunit B

Retrograde neuronal labeling with CTB was performed as described previously [43]. Briefly, 200 nl of 0.2% Alexa Fluor 555-conjugated CTB (Thermo Fisher Scientific) dissolved in D-PBS was unilaterally infused into the lateral septum with the stereotaxic method described in Supplementary Materials and Methods. After two weeks, brain sections were made, stained with Hoechst 33342, and mounted on APS-coated glass slides, and confocal images were acquired, as described in “Immunofluorescent staining”.

### Chemogenetic manipulation and anterograde axonal labeling with viral vectors

Plasmids for AAV-hSyn-DIO-hM4Di-mCherry (Addgene plasmid # 44362 ; http://n2t.net/addgene:44362 ; RRID:Addgene_44362) and AAV-hSyn-DIO-mCherry (Addgene plasmid # 50459 ; http://n2t.net/addgene:50459 ; RRID:Addgene_50459) were gifts from Bryan Roth [44]. Plasmids for AAV-CaMKII-Cre and AAVrg-CaMKII-Cre were generated in our previous study [14]. Conventional or retrograde AAV vector particles were generated as previously described [14]. Briefly, each of the above plasmids was co-transfected into AAV293 cells with a plasmid encoding AAV2/rh10 or AAVrg [45] capsid proteins, respectively, and a pHelper plasmid. The plasmid encoding AAVrg capsid proteins (rAAV2-retro helper) was a gift from Alla Karpova & David Schaffer (Addgene plasmid # 81070; http://n2t.net/addgene:81070 ; RRID:Addgene_81070).

For DREADD-mediated inhibition of excitatory neurons in the piriform cortex and its control, either AAV2/rh10-hSyn-DIO-hM4Di-mCherry (8.0 × 10^11^ vg/ml, 250 nl) or AAV2/rh10-hSyn-DIO-mCherry (3.0 × 10^12^ vg/ml, 250 nl) were bilaterally infused with AAV2/rh10-AAV-CaMKII-Cre (2.3 × 10^13^ vg/ml, 250 nl) into the anterior and posterior piriform cortex using the stereotaxic method described in Supplementary Materials and Methods. For DREADD-mediated activation of excitatory neurons in the piriform cortex and its control, either AAV2/rh10-hSyn-DIO-hM3Dq-mCherry (3.0 × 10^11^ vg/ml, 250 nl) or AAV2/rh10-hSyn-DIO-mCherry (3.0 × 10^12^ vg/ml, 250 nl) were bilaterally infused with AAV2/rh10-AAV-CaMKII-Cre (2.3 × 10^13^ vg/ml, 250 nl) into the anterior and posterior piriform cortex. For DREADD-mediated inhibition of the piriform cortex to lateral septum pathway and its control, either AAV2/rh10-hSyn-DIO-hM4Di-mCherry (8.0 × 10^11^ vg/ml, 500 nl) or AAV2/rh10-hSyn-DIO-mCherry (3.0 × 10^12^ vg/ml, 500 nl) was bilaterally infused into the anterior and posterior piriform cortex, and AAVrg-AAV-CaMKII-Cre (1.2 × 10^13^ vg/ml, 500 nl) was bilaterally infused into the lateral septum. For DREADD-mediated inhibition of the piriform cortex to non-specific thalamic nuclei pathway and its control, either AAV2/rh10-hSyn-DIO-hM4Di-mCherry (8.0 × 10^11^ vg/ml, 500 nl) or AAV2/rh10-hSyn-DIO-mCherry (3.0 × 10^12^ vg/ml, 500 nl) was bilaterally infused into the anterior and posterior piriform cortex, and AAVrg-AAV-CaMKII-Cre (1.2 × 10^13^ vg/ml, 500 nl) was bilaterally infused into the non-specific thalamic nuclei. DREADD-mediated activation of the piriform cortex to lateral septum pathway and its control, either AAV2/rh10-hSyn-DIO-hM3Dq-mCherry (3.0 × 10^11^ vg/ml, 500 nl) or AAV2/rh10-hSyn-DIO-mCherry (3.0 × 10^12^ vg/ml, 500 nl) was bilaterally infused into the anterior and posterior piriform cortex, and AAVrg-AAV-CaMKII-Cre (1.2 × 10^13^ vg/ml, 500 nl) was bilaterally infused into the lateral septum.

After three weeks to allow gene expression from AAV vectors, to activate hM4Di or hM3Dq during SDS, the DREADD ligand clozapine-N-oxide (CNO; Enzo Life Science) dissolved in saline containing 0.5% dimethyl sulfoxide was administered intraperitoneally at 1 mg/kg in a 10 ml/kg volume.

### Statistical analysis

Statistical analyses were performed using Prism 10 (GraphPad Software). Error bars represent SEM. Differences between two groups were analyzed with the two-tailed unpaired Student’s *t*-test. Differences among more than two groups were analyzed with one-way analysis of variance (ANOVA) or two-way ANOVA, depending on the number of factors, followed by Tukey’s or Bonferroni’s multiple comparison test. *P* values were determined to reflect multiple comparisons adjustment, if necessary, and those less than 0.05 were considered statistically significant. The number of samples in each group was determined based on published studies and our previous experience with the behavioral tests employed. No statistical methods were used to predetermine the sample size.

## Results

### Whole brain imaging revealed various effects of acute and chronic stress on brain-wide neural activities

**Fig. 1:**
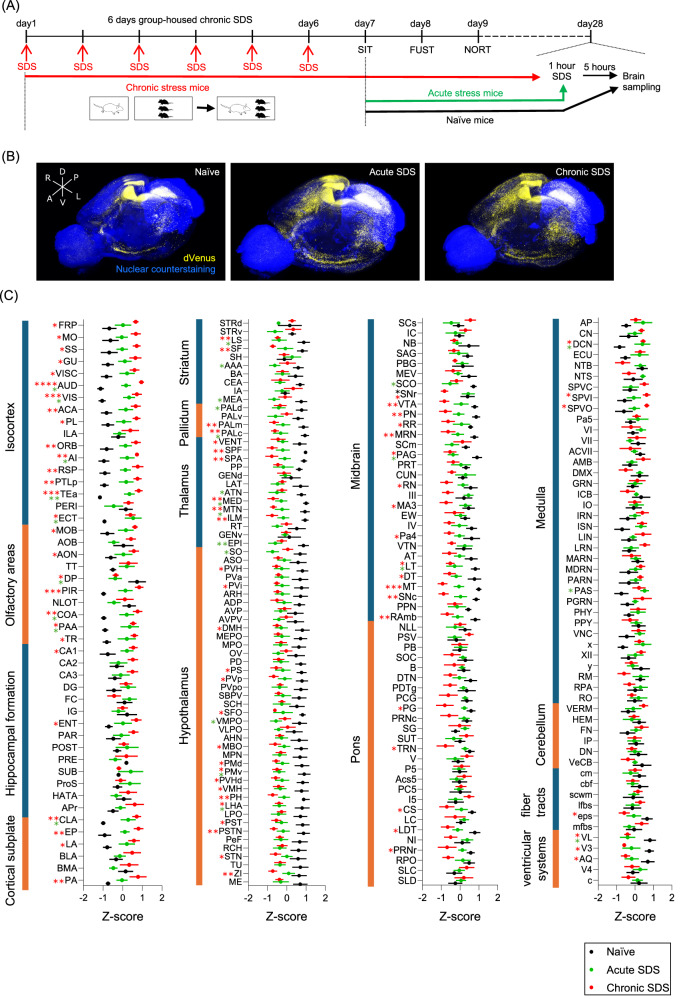
Whole brain imaging revealed the effects of acute and chronic social defeat stress on brain-wide neural activities. **A** Experimental schedule of acute and chronic social defeat stress (SDS), behavioral tests, and whole brain imaging with Arc-dVenus mice. SDS was applied by introducing a male ICR mouse (white) into the home cage of three male Arc-dVenus mice (black). Mice with chronic SDS received SDS for 1 h daily for six consecutive days (Day 1 to Day 6) followed by the social interaction test (SIT, Day 7), female urine sniffing test (FUST, Day 8), and novel object recognition test (NORT, Day 9). After 3 weeks to minimize possible interference from the behavioral tests, the mice received an additional SDS 5 h before sacrifice for whole brain imaging (Day 28). Mice with acute SDS or naïve mice were sacrificed for whole brain imaging with or without SDS, respectively (Day 28), after the behavioral tests (Day 7 to Day 9). **B** Representative whole brain images of Arc-dVenus mice without SDS (Naïve) or with acute or chronic SDS. dVenus fluorescent signals were shown in yellow. Nuclear counterstaining with Hoechst 33342 was shown in blue. A: anterior, P: posterior, D: dorsal, V: ventral, L: left, and R: right. **C** Z-scores of dVenus fluorescent intensities in the brain of Arc-dVenus mice without SDS (Naïve, black circle) or with acute or chronic SDS (green or red circle, respectively). dVenus intensities measured from designated brain regions were normalized to the distribution among individual mice to calculate z-scores. *N* = 7 in each group. Brain regions were grouped into broader brain structures according to the Allen Mouse Brain Atlas. Abbreviations for brain regions are shown in Supplementary Table S1. Two-way ANOVA results are shown in Supplementary Table S3. *P < 0.05, **P < 0.01, ***P < 0.001, ****P < 0.0001 for Tukey’s multiple comparisons test between naïve mice and those with acute or chronic SDS (green or red, respectively) or between those with acute and chronic SDS (black). Error bars represent means ± SEM.

To visualize the brain-wide distribution of neural activities, we subjected adult male Arc-dVenus neural response reporter mice to acute or chronic SDS (Fig. 1A). The mice were sacrificed after the last stress exposure for the whole brain imaging. Naïve mice were kept without any stress exposure before sacrifice (Fig. 1A). Before the last stress exposure, we confirmed multiple behavioral changes induced by chronic SDS. Thus, chronic SDS led to social avoidance from a novel social target in the social interaction test, as measured by the time spent in the interaction or avoidance zone, not affecting general locomotor activity (Supplementary Fig. 1A–D). In the female urine sniffing test, naïve mice preferred female urine, an innate rewarding stimulus, to water, but the time to sniff female urine was selectively reduced in chronic SDS mice, suggesting anhedonia (Supplementary Fig. 1E). In the novel object recognition test (Supplementary Fig. 1F), naïve mice preferred a novel object to a familiar object without spatial bias (Supplementary Fig. 1G). By contrast, the novel object preference was abolished in chronic SDS mice, suggesting cognitive decline (Supplementary Fig. 1H). Given the variability in chronic SDS-induced social avoidance (see Supplementary Fig. 1C) as frequently reported, we categorized these mice into susceptible mice, whose time spent in both the interaction zone and the avoidance zone fell outside the distributions of Naïve mice, and resilient mice, which comprised the remainder (Supplementary Fig. 1I–K). We noted that both susceptible and resilient mice exhibited signs of anhedonia in the female urine sniffing test and cognitive decline in the novel object recognition test, suggesting that stress susceptibility manifests independently across different behavioral domains.

**Fig. 2:**
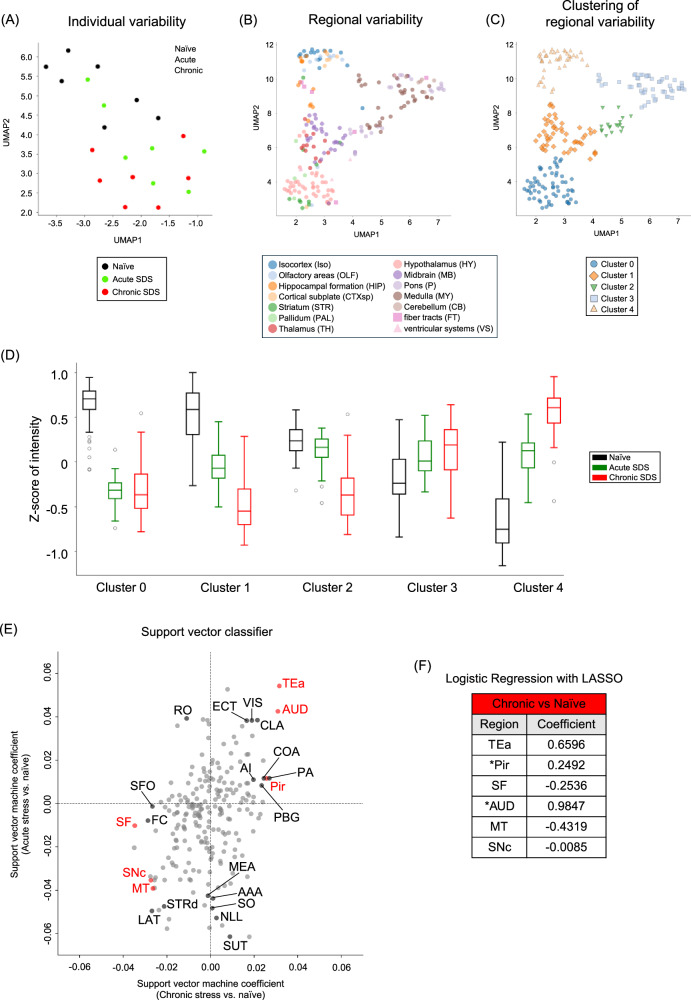
Widely distributed brain regions differently contribute to neural responses to acute and chronic social defeat stress. **A** UMAP plot to visualize the variability of brain-wide neural activities among naïve (black), acute SDS (green), and chronic SDS (red) individual mice, based on fluorescent intensities of Arc-dVenus mice. **B** UMAP plot to visualize the variability of neural activities among 223 brain regions. The color of a data point represents the brain structure which each brain region belongs to, as indicated below. **C** Five clusters of brain regions defined with UMAP clustering analysis. **D** Box plots of z-scores of neural activities in brain regions categorized to the indicated clusters defined in (**C**) for naïve (black), acute SDS (green), and chronic SDS (red) mice. Each box shows the interquartile range (IQR) with the median in the middle. The whiskers cover the datapoints outside, except outliers that were distant from the IQR by more than its 1.5 times. **E** Plots of support vector machine coefficients for respective brain regions to classify neural activities in acute SDS or chronic SDS mice from those in naïve mice in the vertical or horizontal axis, respectively. **F** Brain regions whose contributions were selected by LASSO analysis to classify neural activities in chronic SDS mice from those in naïve mice and their LASSO coefficients. Asterisks indicate sensory cortices (i.e., Pir and AUD). These brain regions are highlighted in red in (**E**). Abbreviations for brain areas are shown in Supplementary Table S1.

We then acquired whole-brain fluorescent images from Arc-dVenus mice and parcellated them into 223 regions from 14 structures, based on the Allen Mouse Brain Atlas (Fig. 1B, Supplementary Table 1) [39]. Mean dVenus fluorescent intensity in each region was defined as its regional activity. Given large variability among brain regions, we transformed regional activities into z-scores to represent individual variability within each region (Fig, 1C, Supplementary Fig. 2). We first performed dimensionality reduction with uniform manifold approximation and projection (UMAP) to examine the variability in regional activities among individual mice and brain regions (Fig. 2A, B). Regional activities of chronic SDS mice were separated from those of naïve mice, although those of acute SDS mice were in between, not clearly separated from either group of mice (Fig. 2A). Regional activities of respective brain regions were separated into five clusters with each cluster having unique patterns (Fig. 2C, D, Supplementary Table S2). According to the definition of brain structures in the Allen Mouse Brain Atlas, Cluster 0 regions, from striatum and hypothalamus, showed rapid decrease after acute SDS being maintained after chronic SDS. Cluster 1 regions, mostly from thalamus and midbrain, showed gradual decrease after acute and chronic SDS. Cluster 2 regions, mostly from medulla and pons, showed decrease only after chronic SDS. Cluster 3 regions, mostly from pons, medulla, and cerebellum, responded to neither acute nor chronic SDS. Cluster 4 regions, mostly from isocortex, olfactory areas, hippocampal formation, and cortical subplate, showed gradual increase after acute and chronic SDS. Notably, neural responses of sensory and motor cortices were similar to those of association cortices, although the latter regions have been studied more extensively than the former in the context of stress.

To relate regional activities of chronic SDS mice to stress susceptibility, we compared the Arc-dVenus signals between susceptible and resilient mice across various brain regions (Supplementary Fig. 3). Some brain regions showed similar responses to chronic SDS between susceptible and resilient mice, whereas other brain regions showed preferential responses to chronic SDS in either susceptible or resilient mice, compared with Naïve mice. To examine the brain-wide distribution of responses to chronic SDS in susceptible and resilient mice, we separately analyzed UMAP-based clusters of regional activity for each group to capture the variability among individual mice and brain regions (Supplementary Fig. 4A). This analysis revealed a moderate distinction between susceptible and resilient mice. Additionally, a distinct preference to either susceptible or resilient mice was observed among the five clusters described above (Supplementary Fig. 4B). Thus, cluster 0 regions, from striatum and hypothalamus, showed a decrease in activity in both susceptible and resilient mice, as well as in acute SDS mice, with a preference toward susceptible mice. Cluster 1 regions, mostly from thalamus and midbrain, showed a decrease in activity in both groups and in acute SDS mice, with a preference toward resilient mice. Cluster 2 regions, mostly from medulla and pons, showed a selective decrease in resilient mice without changes in acute SDS mice. Cluster 3 regions, mostly from pons, medulla, and cerebellum, showed a selective increase in susceptible mice without changes in acute SDS mice. Cluster 4 regions, mostly from isocortex, olfactory areas, hippocampal formation, and cortical subplate showed an increase in activity in both susceptible and resilient mice, as well as in acute SDS mice, with a preference toward resilient mice. These findings suggest that chronic stress recruits distinct neural circuits that are selectively active in either susceptible or resilient mice, which are not responsive to acute stress. By contrast, brain regions responsive to both acute and chronic stress exhibited activity in both susceptible and resilient mice, albeit with some preference for one group over the other.

### Widely distributed brain regions differently contribute to neural responses to stress

To identify the brain regions that contribute to classifying neural activities in acute SDS or chronic SDS mice from those in naïve mice, we analyzed brain-wide regional activities using a support vector machine (SVM). As conventionally performed, we evaluated the contribution of each brain region in the SVM classifier with the value of its coefficient [40, 46]. Some brain regions contributed to both acute and chronic SDS (e.g., temporal association cortex (TEa), claustrum (CLA), auditory cortex (AUD), visual cortex (VIS), and ectorhinal cortex (ECT) for positive coefficients; dorsal striatum (STRd), lateral group of the dorsal thalamus (LAT), and substantia nigra pars compacta (SNc) for negative coefficients) (Fig. 2E). Other brain regions selectively contributed for either acute SDS (e.g., raphe nucleus obscurus (RO) for positive coefficients; medial amygdala (MEA), anterior amygdaloid area (AAA), supraoptic nucleus (SO), nucleus of the lateral lemniscus (NLL), and supratrigeminal nucleus (SUT) for negative coefficients) or chronic SDS (e.g., piriform cortex (Pir), anterior insular cortex (AI), posterior amygdala (PA), cortical amygdaloid nucleus (COA), parabigeminal nucleus (PBG) for positive coefficients; septofimbrial nucleus (SF), fasciola cinereum (FC), subfornical organ (SFO) for negative coefficients) (Fig. 2E).

Notably, multiple sensory cortices have stronger contributions than stress-related association cortices, such as prelimbic and infralimbic cortices. Auditory and visual cortices contributed for both acute and chronic SDS, whereas piriform (olfactory) and gustatory cortices contributed selectively for chronic SDS. Somatosensory cortex did not contribute for acute nor chronic SDS. The contributions of auditory and piriform cortices for chronic SDS were substantiated with logistic regression with the least absolute shrinkage and selection operator (LASSO) (Fig. 2F), an L1 regularization widely used for dimensionality reduction and feature selection [41].

These findings demonstrate that widely distributed brain regions differently contribute to neural responses to acute and chronic stress, suggesting acute and chronic stress-selective bias in the activation of multiple neural pathways, including those for sensory processing.

### The activation of excitatory neurons in the piriform cortex during chronic stress is crucial for the induction of behavioral disturbance

**Fig. 3:**
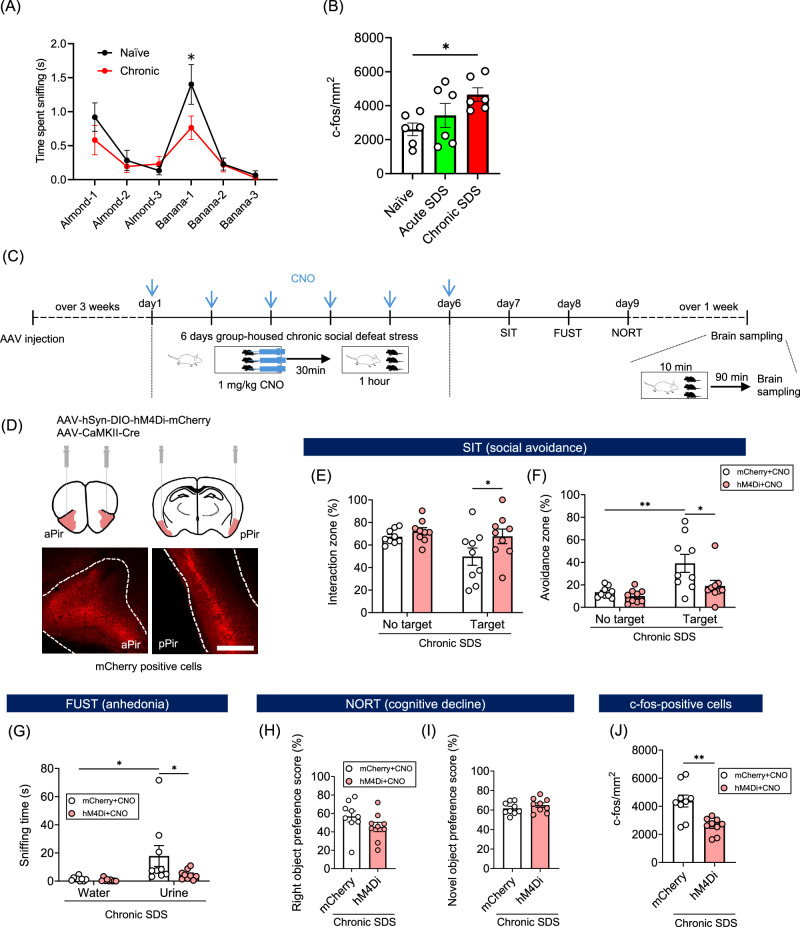
The activation of excitatory neurons in the piriform cortex during chronic social defeat stress is crucial for the induction of behavioral disturbance. **A** Odor discrimination deficit after chronic SDS. Naïve mice (black) or chronic SDS mice (red) were presented with either almond or banana extract for 2 min, with the same odor repeated three times (i.e., Almond-1 to Almond-3 and Banana-1 to Banana-3). The time spent sniffing odorants during each session for 2 min was plotted. The increase in the sniffing time upon odor switching is considered as a behavioral index of odor discrimination. *N* = 9 for naïve mice, and *N* = 11 for chronic SDS mice. *P < 0.05 for Bonferroni’s multiple comparisons test. Error bars represent means ± SEM. **B** The density of c-Fos-positive cells in the piriform cortex of naïve, acute SDS, and chronic SDS mice. *N* = 6 in each group. *P < 0.05 for Bonferroni’s multiple comparisons test. Error bars represent means ± SEM. **C** Experimental schedule for DREADD-mediated inhibition of excitatory neurons in the piriform cortex during chronic SDS and subsequent behavioral tests. Three weeks after AAV vector injection, mice were exposed to SDS 30 min after daily CNO administration for 6 days (blue arrows, Day 1 to Day 6). After 24 h recovery, the social interaction test (SIT, Day 7), female urine sniffing test (FUST, Day 8), and novel object recognition test (NORT, Day 9) were conducted without CNO administration. After 1 week or more for recovery, mice received an additional SDS before brain sampling for c-Fos immunofluorescent staining. **D** AAV vector injection for DREADD-mediated inhibition of excitatory neurons in the piriform cortex. AAV-hSyn-DIO-hM4Di-mCherry was stereotaxically injected with AAV-CaMKII-Cre into anterior and posterior piriform cortices (aPir and pPir, respectively; upper panels). Representative images of mCherry-positive cells in these regions are shown (lower panels). Scale bar, 200 μm. **E**–**I** Results of the behavioral tests. Mice expressing mCherry alone or hM4Di-mCherry were subjected to the behavioral tests as shown in (**C**). In the social interaction test (SIT), the proportion of the time spent in the interaction zone (**E**) and the avoidance zone (**F**) without or with a target ICR mouse (No target or Target, respectively) was used to determine the level of social avoidance. In the female urine sniffing test (FUST), the time spent sniffing water or female urine was used to determine the level of anhedonia (**G**). In the novel object recognition test (NORT), the proportion of the time spent sniffing the right object during the first session (**H**) and the proportion of the time spent sniffing the novel object during the second session (**I**) were used to determine the level of spatial bias and cognitive decline, respectively. *N* = 9 in each group. **J** The effect of DREADD-mediated inhibition on SDS-induced c-Fos expression in the piriform cortex. Mice expressing mCherry alone or hM4Di-mCherry were subjected to c-Fos immunofluorescent staining as shown in (**C**). The density of c-Fos-positive cells in the piriform cortex is shown. *N* = 9 in each group. One-way ANOVA and two-way repeated measures ANOVA results are shown in Supplementary Table S3. *P < 0.05, **P < 0.01 for Bonferroni’s multiple comparisons test (**E**–**G**). **P < 0.01 for unpaired *t*-test (**J**). Error bars represent means ± SEM.

Given primary contribution of the piriform cortex to classifying neural responses to chronic SDS, we examined whether olfactory processing per se might be altered following chronic SDS in C57BL/6 mice. Naïve mice demonstrated the ability to detect a change in odor, whereas chronic SDS mice did not (Fig. 3A), suggesting olfactory dysfunction. Immunofluorescent staining for c-Fos, conducted with a separate cohort of mice from those used for whole-brain imaging, further confirmed that neurons in the piriform cortex responded to chronic SDS more strongly than acute SDS (Fig. 3B, Supplementary Fig. 5A). We also examined c-Fos-positive cells separately in the anterior and posterior regions of the piriform cortex, since these regions have anatomical and functional differences [47]. The density of c-Fos-positive cells was higher in the anterior region than in the posterior region, regardless of stress exposure (Supplementary Fig. 5B, C). Chronic SDS increased the density of c-Fos-positive cells in both the anterior and posterior regions, although statistical significance was achieved only in the anterior region. Using designer receptors exclusively activated by designer drugs (DREADD), we tested whether the activation of the piriform cortex during chronic SDS is required for the induction of behavioral disturbance. Since most neurons in the piriform cortex are excitatory projection neurons [48], we expressed the Gi-coupled inhibitory DREADD (hM4Di) in piriform excitatory neurons of adult male mice by bilateral infusion into the piriform cortex of an AAV vector expressing either hM4Di fused with mCherry (hM4Di-mCherry) in a Cre recombinase-dependent manner with an AAV expressing Cre recombinase under the Camk2α promoter (Fig. 3C, D). As control, we expressed mCherry alone instead of hM4Di-mCherry. After the three-week interval, the mice expressing hM4Di-mCherry or mCherry alone in piriform excitatory neurons were subjected to chronic SDS. The mice were administered with CNO, a DREADD ligand, 30 min before each stress exposure to inhibit piriform excitatory neurons during SDS and subjected to multiple behavioral tests (Fig. 3C). It should be noted that DREADD-mediated inhibition was not applied during the behavioral tests. In the social interaction test, DREADD-mediated inhibition of piriform excitatory neurons during chronic SDS abolished the induction of social avoidance (Fig. 3E, F), reducing the proportion of susceptible mice (11% for hM4Di and 44% for control). In the female urine sniffing test and novel object recognition test, no effects of DREADD-mediated inhibition were observed (Fig. 3G-I). After these behavioral tests, we confirmed that DREADD-mediated inhibition of piriform excitatory neurons reduced chronic SDS-induced c-Fos expression (Fig. 3J). We also tested the effects of DREADD-mediated activation of piriform excitatory neurons with Gq-coupled excitatory DREADD (hM3Dq) (Supplementary Fig. 6A). Surprisingly, the activation of the piriform cortex combined with acute SDS immediately made 3 out of 5 mice immobile, leading to their death on the next day (Supplementary Fig. 6B). By contrast, DREADD-mediated activation of the piriform cortex alone, without SDS, did not induce any behavioral changes nor result in death (Supplementary Fig. 6C–J). These results demonstrate that activation of excitatory neurons in the piriform cortex during chronic stress is necessary, though not sufficient, for the induction of social avoidance. Excessive activation of the piriform cortex appears to exacerbate the effects of SDS, potentially leading to fatal outcomes.

### The piriform cortex directly projects to and activates the lateral septum in response to chronic stress

**Fig. 4:**
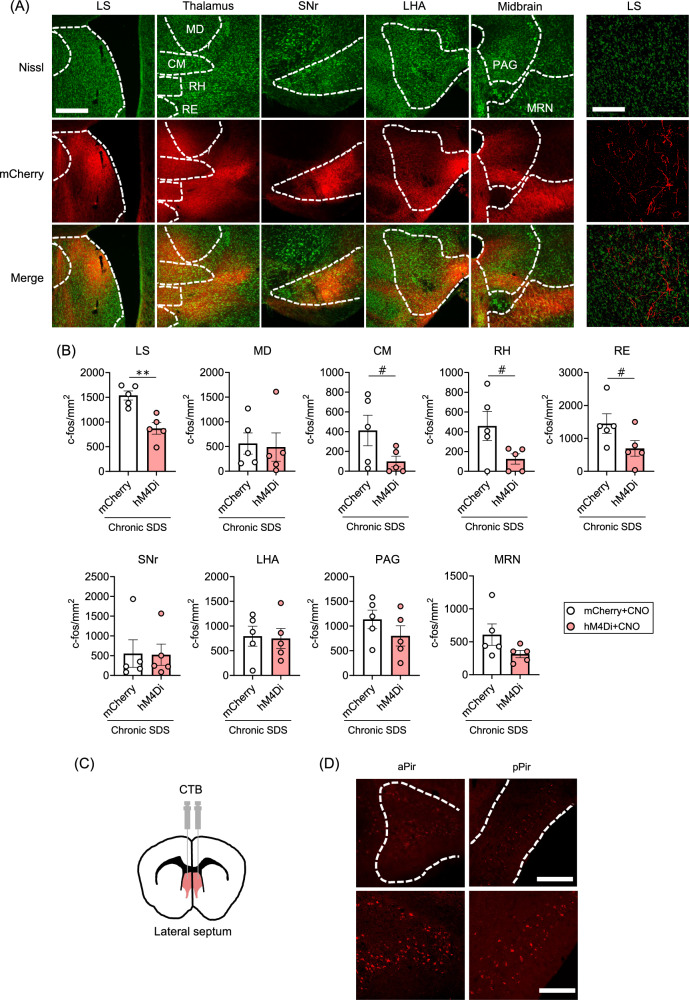
Excitatory neurons in the piriform cortex project directly to the lateral septum and activate it in response to chronic social defeat stress. **A** Representative images of mCherry-positive axons of excitatory neurons in the piriform cortex. The same mice expressing mCherry alone used for chronic SDS and the behavioral tests shown in Fig. 3 were sacrificed for immunofluorescent staining for mCherry. Fluorescent Nissl and mCherry signals are shown in green and red, respectively. Dotted lines indicate brain region boundaries. Scale bars, 200 μm. Magnified images of the lateral septum are also shown (right panels). Scale bars, 100 μm. **B** Effects of DREADD-mediated inhibition of excitatory neurons in the piriform cortex on SDS-induced c-Fos expression in the brain regions shown in (**A**). The same mice used in Fig. 3J, expressing mCherry alone or hM4Di-mCherry in excitatory neurons in the piriform cortex and administered with CNO during chronic SDS (mCherry+CNO or hM4Di+CNO, respectively), were used. *N* = 5 in each group. #*P* < 0.1, **P < 0.01 for unpaired *t*-test. Error bars represent means ± SEM. **C**, **D** Retrograde labeling of piriform neurons projecting to the lateral septum with CTB. Fluorescently labelled CTB was stereotaxically injected into the lateral septum, highlighted in red (**C**). Representative images of CTB-positive cells in anterior and posterior piriform cortices (**D**, aPir and pPir, respectively). Magnified images are also shown (**D**, lower panels). Scale bar, 200 μm (upper panels) and 100 μm (lower panels). Abbreviations for brain regions are shown in Supplementary Table 1.

To identify the brain regions that receive axons from excitatory neurons in the piriform cortex, we sacrificed the mice expressing mCherry in these neurons after chronic SDS and the behavioral tests and visualized mCherry-positive axons throughout the brain (Fig. 4A). We excluded adjacent brain regions to the injection site, where extensive fluorescent signals prevented us from recognizing axonal projections. mCherry-positive axons were observed in various brain regions, such as the lateral septum, mediodorsal nucleus of thalamus, centromedial nucleus of thalamus, rhomboid nucleus, nucleus of reuniens, substantia nigra pars reticulata, lateral hypothalamic area, periaqueductal gray, and midbrain reticular nucleus (Fig. 4A). Next, we examined whether these brain regions were activated by the piriform cortex in response to chronic SDS (Fig. 4B). DREADD-mediated inhibition of piriform excitatory neurons reduced c-Fos-positive neurons in the lateral septum and non-specific thalamic nuclei (i.e., centromedial nucleus of thalamus, rhomboid nucleus, and nucleus of reuniens), although only the former showed statistical significance (Fig. 4B). We retrogradely labelled neurons that send axons to the lateral septum with fluorescent cholera toxin subunit B and confirmed direct projection from the piriform cortex to the lateral septum (Fig. 4C, D). These results demonstrate that the piriform cortex directly projects to and activates the lateral septum in response to chronic SDS. Since the expression of activity markers in the piriform cortex increased with repetition of SDS, we examined whether chronic SDS also affected axonal projections from the piriform cortex to the lateral septum. As described above, chronic SDS mice showed strong mCherry signals derived from axons of piriform excitatory neurons in the lateral septum, particularly enriched in its dorsolateral part (Supplementary Fig. 7A). By contrast, Naïve mice displayed weaker mCherry signals in the lateral septum, possibility due to lower mCherry expression in the piriform cortex compared to chronic SDS mice (Supplementary Fig. 7B). Nonetheless, the distribution of mCherry signals in the lateral septum appeared to differ between the groups, with Naïve mice showing no clear enrichment in the dorsolateral part. This finding suggests that chronic SDS induces structural plasticity in the piriform cortex to lateral septum pathway.

### The activation of the piriform cortex to lateral septum pathway during chronic stress is crucial for the induction of behavioral disturbance

**Fig. 5:**
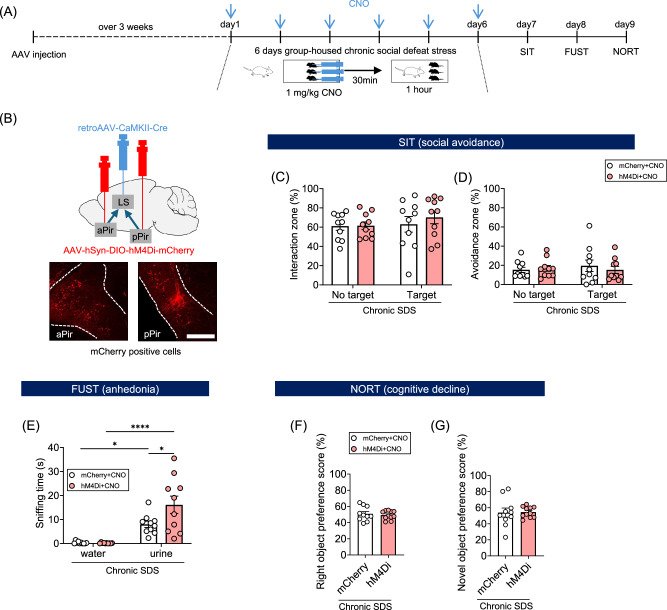
The activation of the piriform cortex to lateral septum pathway during chronic social defeat stress is crucial for the induction of behavioral disturbance. **A** Experimental schedule for DREADD-mediated inhibition of the piriform cortex to lateral septum pathway during chronic SDS and subsequent behavioral tests. The experiments were scheduled as described in the legend of Fig. 3C. **B** AAV vector injection for DREADD-mediated inhibition of excitatory neurons in the piriform cortex projecting to the lateral septum. AAV-hSyn-DIO-hM4Di-mCherry and retroAAV-CaMKII-Cre were stereotaxically injected into anterior and posterior piriform cortices (aPir and pPir, respectively) and the lateral septum (LS), respectively (upper panel). Representative images of mCherry-positive cells in the aPir and pPir are shown (lower panels). Scale bar, 200 μm. **C**–**G** Results of the behavioral tests. Mice expressing mCherry alone or hM4Di-mCherry in the targeted neurons and administered with CNO during chronic SDS (mCherry+CNO or hM4Di+CNO, respectively) were subjected to the behavioral tests as shown in (**A**). Behavioral indices shown in these graphs are described in the legend of Fig. 3E–I. *N* = 10 for mCherry alone, *N* = 10 for hM4Di-mCherry. Two-way repeated measures ANOVA results are shown in Supplementary Table S3. *P < 0.05, ****P < 0.0001 for Bonferroni’s multiple comparisons test. Error bars represent means ± SEM.

We examined whether the activation of the piriform cortex to lateral septum pathway during chronic SDS is involved in the induction of behavioral disturbance. To selectively inhibit these neurons, we infused a retrograde AAV vector expressing Cre recombinase under the CamkIIα promoter into the lateral septum and a conventional AAV vector expressing hM4Di-mCherry or mCherry alone in the presence of Cre recombinase into the piriform cortex (Fig. 5A, B). Then the mice were subjected to chronic SDS with CNO administered 30 min before each stress exposure (Fig. 5A). In the female urine sniffing test, DREADD-mediated inhibition of the piriform cortex to lateral septum pathway during chronic SDS increased the female urine sniffing time, suggesting the loss of chronic stress-induced anhedonia (Fig. 5E). In the social interaction test, the same DREADD-mediated inhibition appeared to shorten the time for the social avoidance zone, although it did not reach statistical significance (Fig. 5C, D). No effects were observed in the novel object recognition test (Fig. 5F, G). On the other hand, DREADD-mediated inhibition of the piriform cortex to non-specific thalamic nuclei pathway did not affect any of these behavioral changes (Supplementary Fig. 8). In contrast to DREADD-mediated inhibition during chronic SDS, DREADD-mediated activation of the piriform cortex to lateral septum pathway alone, without SDS, did not induce any behavioral changes (Supplementary Fig. 9). These results demonstrate that the activation of the piriform cortex to lateral septum pathway during chronic stress is necessary, though not sufficient, for the induction of behavioral disturbance.

## Discussion

Neural pathways that process external stimuli as stress to cause behavioral disturbance remains underexplored. Using the whole brain imaging of Arc-dVenus neuronal response reporter mice, we discovered that widely distributed brain regions differently contribute to neural responses to acute and chronic SDS. Machine learning analysis showed that multiple sensory cortices contribute to neural responses to acute and chronic SDS more strongly than stress-related association cortices, such as prelimibic and infralimbic cortices. The contributions of sensory cortices for acute and chronic SDS varied depending on the sensory modality. Whereas auditory and visual cortices contributed for both acute and chronic SDS, olfactory (piriform) and gustatory (insular) cortices did selectively for chronic SDS. Indeed, the piriform cortex response to SDS was augmented with repetition of stress, accompanied by impaired odor discrimination. Furthermore, the activation of the piriform cortex to lateral septum pathway during chronic SDS was crucial for the induction of behavioral disturbance. These findings highlight the importance of the olfactory pathway in perceiving chronic stress to cause behavioral disturbance via its connection to the lateral septum.

Whole brain imaging with Arc-dVenus reporter mice revealed at least five patterns of regional responses to acute and chronic SDS. These include early decrease after acute SDS being maintained after chronic SDS in the hypothalamus and striatum; gradual decrease after acute and chronic SDS in the midbrain and thalamus; gradual increase after acute and chronic SDS in the isocortex, olfactory areas, hippocampus, and cortical subplates; and late decrease only after chronic SDS in the medulla and pons. Thus, there is a spatially defined sequence of neural consequences of stress: the hypothalamus and striatum are initially affected after acute SDS; more brain structures, including corticothalamic loops, are gradually affected; and the midbrain and pons remain spared until chronic SDS. Whether a given pattern of neural responses in multiple brain regions is generated by a specific brain region as a hub or through diffuse projections and whether different patterns of neural responses are causally related with each other warrant to be investigated.

With Arc-dVenus reporter mice, whereas most cerebral cortices similarly showed gradual signal increase with repetition of stress, some cortical regions, especially multiple sensory cortices, have greater increase than the others. Indeed, clinical and preclinical studies have shown the association of olfactory, visual, and auditory dysfunctions with stress and depression [17–31]. However, no studies so far have explored how sensory pathways are engaged in perceiving chronic stress and related to the induction of behavioral disturbance. Differential contributions of each brain region to neural responses to acute and chronic SDS led us to propose that each sensory pathway plays a distinct role in stress perception: The visual and auditory pathways are employed to detect a stressor regardless of acute and chronic SDS, whereas the olfactory pathway is recruited with chronic SDS to detect a stressor more effectively. However, this adaptive change with chronic SDS could negatively affect emotional behaviors, since the olfactory pathway is directly connected with the emotional neural circuit [49–53], bypassing the thalamus, unlike the visual and auditory pathways [54–56].

Our findings showed that the activation of the piriform cortex during stress perception causes behavioral disturbance via its connection to the lateral septum. This finding is consistent with the notion that the activation of the lateral septum, especially neurotensin-expressing neurons, underlies social anhedonia [57]. These neurons reportedly become responsive to a social target only when mice display social anhedonia after chronic SDS [57]. The social sensitivity of these neurons may result from a chronic SDS-induced alteration in axonal projections from the piriform cortex to the lateral septum, as suggested by this study. Since the lateral septum does not receive direct inputs from other sensory pathways [58–61], the recruitment of the piriform cortex with chronic stress could be essential for social stimuli to activate the lateral septum for behavioral disturbance. The inputs from the piriform cortex could enable the lateral septum to associate olfactory social cues with behavioral context and emotional valence, each of which can be informed from the hippocampus and the amygdala, respectively. It should be noted that the lateral septum responds not only to social stress but also to non-social stressors, such as electric foot shocks and restraint stress [59, 62]. Similarly, the piriform cortex is activated by a wide range of stressors that are neither olfactory nor social, including restraint stress and chronic corticosterone treatment [63]. Therefore, the piriform cortex to the lateral septum pathway may play a common role in various types of stress, although this hypothesis requires further investigation.

Given the presence of olfactory dysfunction in a variety of psychiatric and neurological disorders, the olfactory pathway identified in this study may be involved beyond diagnostic boundaries. However, since the chronic SDS model used in this study involves social recognition that relies more heavily on olfaction in rodents than in humans, caution is warranted when extrapolating the significance of the olfactory pathway to stress-related mental illnesses. Nonetheless, recent clinical and preclinical studies suggested the importance of other sensory modalities in mental illness. In addition, non-olfactory stimuli can also be perceived as stress to induce behavioral disturbance and risk having mental illnesses. Therefore, the roles of multiple sensory pathways in chronic stress pathology in mental illness warrant future investigation.

There are several limitations of this study. First, it should be noted that chronic SDS model used in this study is a modified version that involves group housing for the defeated C57BL/6 mice, in contrast to the classical SDS model, where defeated mice are housed in isolation and applied to SDS in the home cage of intruder ICR mice. While group-housed SDS allows for the processing of more mice, it may introduce potential confounding factors by influencing home cage behaviors. To address this, we monitored fighting and allogrooming behaviors, two key indices of agonistic social encounters, in the home cage during a one-hour period before and after each SDS exposure. While allogrooming increased during the one-hour period before SDS (but not after), the frequency remained low at 1–2 times per hour (Supplementary Fig. 10). Additionally, no fighting was observed in any observation period. Therefore, we concluded that chronic SDS did not significantly disrupt home cage behaviors. However, since we did not conduct comprehensive behavioral analyses in the home cage due to their time-consuming nature, we cannot entirely rule out subtle effects of SDS on home cage behaviors that might contribute to the differences observed between the groups in our study. In addition, social support or familiarity with cage mates may mitigate the effects of SDS [64, 65]. Second, since we used the group-housed SDS protocol in this study, we could not determine the level of aggression that each mouse received to correlate it to chronic SDS-induced behavioral and neuronal changes. Nonetheless, we manually counted the total number of attacks in each cage and found no correlation with any behavioral changes nor the overall chronic SDS-induced changes in Arc-dVenus signals, as observed in the average of the three defeated mice per cage (Supplementary Fig. 11). Third, we only analyzed male mice in this study, since chronic SDS relies on territorial aggression between males. Given the reported sex differences in the pathology of stress-related mental illnesses, such as depression and PTSD [7], it is essential to investigate the brain-wide responses to both acute and chronic stress and the behavioral role of the piriform cortex to lateral septum pathway in female mice using stress models applicable to both sexes.

## Supplementary information

Supplementary Materials and Methods and Supplementary Figures

Supplementary Table 1

Supplementary Table 2

Supplementary Table 3
