# Supplementary material for: The activation of the piriform cortex to lateral septum pathway during chronic social defeat stress is crucial for the induction of behavioral disturbance in mice
Source: Neuropsychopharmacology. 2024 Dec 5;50(5):828–40. doi: 10.1038/s41386-024-02034-7 (PMC11914691; doi:10.1038/s41386-024-02034-7)
Supplement: Supplementary file 1 — Supplementary Materials and Methods and Supplementary Figures [file 41386_2024_2034_MOESM1_ESM.pdf]

Supplementary Materials

The activation of the piriform cortex to lateral septum pathway during chronic social defeat stress is crucial for the induction of behavioral disturbance in mice

Yuki Okuda[1], Dongrui Li[1], Yuzuki Maruyama[1], Hirokazu Sonobe[1], Tomoyuki Mano[2], Kazuki Tainaka[3], Ryota Shinohara[1], Tomoyuki Furuyashiki[1]

[1]Division of Pharmacology, Kobe University Graduate School of Medicine, Kobe, 650-0017, Japan
[2]Computational Neuroethology Unit, Okinawa Institute of Science and Technology (OIST) Graduate University, Okinawa, 904-0412, Japan
[3]Department of System Pathology for Neurological Disorders, Brain Research Institute, Niigata University, Niigata, 951-8585, Japan

Correspondence:
Ryota Shinohara, Ph.D.
Division of Pharmacology, Graduate School of Medicine, Kobe University
7-5-1 Kusunoki-cho, Chuo-ku, Kobe, 650-0017, Japan
rshino@med.kobe-u.ac.jp

Tomoyuki Furuyashiki, M.D., Ph.D.
Division of Pharmacology, Graduate School of Medicine, Kobe University
7-5-1 Kusunoki-cho, Chuo-ku, Kobe, 650-0017, Japan
tfuruya@med.kobe-u.ac.jp

Lead Contact: Tomoyuki Furuyashiki, M.D., Ph.D. (tfuruya@med.kobe-u.ac.jp)

Supplementary Materials and Methods

Tissue clearing for whole brain imaging

Anesthetized with pentobarbital (100 mg/kg, Nacalai Tesque, Kyoto, Japan) 5 h after SDS, Arc-dVenus mice were transcardially flushed with ice-cold Dulbecco's modified phosphate-buffered saline (D-PBS; 137 mM NaCl, 2.7 mM KCl, 8.1 mM Na_2HPO_4, and 1.47 mM KH_2PO_4, pH 7.4) and perfused with 4% paraformaldehyde dissolved in 100 mM sodium phosphate buffer (PB, pH 7.4). After being post-fixed for 24 h at 4°C and washed with D-PBS for 2 h each three times, the brains were cleared for whole brain imaging as previously reported [1,2], with some modifications. To preserve dVenus fluorescence and tissue osmolarity, the brains were immersed with shaking in an aqueous solution containing 10% (v/v) 1,2-hexanediol (H0688, Tokyo Chemical Industry), 5 wt% Triton X-100 (12967-45, Nacalai Tesque), 10 mM N-butyldiethanolamine (B0725, Tokyo Chemical Industry), and 1 mM EDTA (E0084, Tokyo Chemical Industry) at 37°C for 3 days. After washing three times with PBS at room temperature (RT) for 30 min each, the brains were stained with TO-PRO-3 Iodide (1:1000 dilution, T3605, Thermo Fisher Scientific) in PBS containing 500 mM NaCl and 10% (v/v) EtOH, with shaking at RT for 7 days. After washing three times with PBS at RT for 30 min each, the samples were immersed in a clearing reagent diluted 1:1 with water and gently shaken at RT for 8 h. The clearing reagent (refractive index = 1.523) was composed of 100 ml Iohexol 350 (Hikari Pharmaceutical), 100 g Nycodenz (18003, Serumwerk Bernburg), and 40 g urea (35904-45, Nacalai Tesque). The brains were then immersed in the clearing reagent with gentle shaking at RT for 1-2 days.

Whole brain images were acquired with a custom-built LSF microscope (MVX10-LS, Evident, Japan). Images were acquired using a 0.63x N.A. 0.15 objective lens (working distance = 87 mm) with an optical zoom of 1.6x. The microscope was equipped with lasers emitting at 488 nm, 532 nm, 594 nm, and 637 nm. A two-dimensional light sheet was formed by a hyperbolic sheet of illumination with these lasers through an illumination lens. The detection objective lens was moved to the axial direction synchronously with the light sheet to avoid defocusing. A brain tissue sample, whose refractive index (RI) was adjusted to 1.523, was immersed in an oil mixture (RI = 1.525) composed of silicon oil HIVAC-F4 (RI = 1.555, Shin-Etsu Chemical) and mineral oil (RI = 1.467, M8410, Sigma-Aldrich) during image acquisition.

Arc-dVenus signals were processed for subsequent analysis according to an established protocol [1,2], as described below. Binary masks created from dVenus-expressing cells were reconstructed into 3D images. These images were parcellated into brain structures and regions, according to the Allen Mouse Brain Atlas [3]. The voxel-standardized intensity within each region was analyzed.

For the computational analysis of the acquired whole brain images, a dVenus fluorescent intensity of each brain region was normalized to the total fluorescent intensities of all measured regions and converted to a z-score based on the variability among individual mice as an index of neural activity. To analyze the distribution of brain-wide neural activities among individual mice or brain regions, dimensionality reduction using Uniform Manifold Approximation and Projection (UMAP) was used with the 'UMAP' function from the Python umap library. UMAP clustering was performed to categorize brain regions according to their pattern of neural activity. To determine the contribution of each brain region to classify neural activities in acute SDS or chronic SDS

mice from those in naïve mice, a support vector machine with a linear kernel was applied to the calculated z-scores of all measured regions, using the 'svm' function from the Python scikit-learn library [4]. Hyperparameters for the support vector machine were optimized using the 'GridSearchCV' function. To identify feature brain regions that primarily contribute to the classification, logistic regression with LASSO (L1 norm regularization) [5] was performed using the 'LogisticRegression' function in the scikit-learn library [4].

Immunofluorescent staining

Immunofluorescent staining was performed as previously described [6]. Anesthetized with sodium pentobarbital (50 mg/kg; Nacalai), mice were transcardially flushed with ice-cold D-PBS and perfused with 4% paraformaldehyde dissolved in 100 mM PB. For c-Fos immunostaining, the mice were subjected to this procedure 90 min after SDS. The brains were post-fixed for 24 h at 4°C, cryoprotected in 30% sucrose in D-PBS for at least 8 h at 4°C, and then coronally sectioned at 30-μm thickness by cryostat. After washes in D-PBS, brain sections were incubated in blocking buffer (0.3% Triton X-100, 1% normal donkey serum in D-PBS) for 1 h at RT and then incubated with primary antibodies in blocking buffer for 1-2 overnights at 4°C. Primary antibodies used in this study are rabbit anti-c-Fos (1:500 dilution; sc-52, Santa Cruz Biotechnology), rabbit anti-mCherry (1:1000 dilution; 600-401-379, Rockland Immunochemicals), and rat anti-GFP (1:500 dilution; GF090R, Nacalai). After washes in D-PBS containing 0.3% Triton-X100 (D-PBS-T) for 10 min each three times, the sections were incubated with Alexa Fluor-conjugated secondary antibodies (1:1000 dilution; Thermo Fisher Scientific) in blocking buffer for overnight at 4°C. After washes with D-PBS-T for 10 min each three times, the sections were incubated with NeuroTrace, a fluorescent Nissl tracer (1:300 dilution; N21480, Thermo Fisher Scientific) in D-PBS for 20 min. After washes in D-PBS for 5min each twice, the sections were incubated with Hoechst 33342 (1:5000 diultion; Thermo Fisher Scientific) in D-PBS for 15 min at RT. After washes for 5 min each twice, the sections were attached on APS-coated glass slides (Matsunami), dried, and mounted in ProLong Gold Antifade Mountant (P36930, Thermo Fisher Scientific).

Confocal images were acquired through a 10x 0.45 N.A. Plan-Apochromat dry objective lens attached to a LSM700 confocal laser microscope (Carl Zeiss) at multiple z-planes and then projected vertically. The densities of c-Fos labeled cells were analyzed using the AnalyzeParticles function in Image J software (https://imagej.nih.gov/ij/).

References for Supplementary Materials and Methods

1. Susaki EA, Tainaka K, Perrin D, Kishino F, Tawara T, Watanabe TM, et al. Whole-brain imaging with single-cell resolution using chemical cocktails and computational analysis. Cell. 2014;157: 726–739.

2. Susaki EA, Tainaka K, Perrin D, Yukinaga H, Kuno A, Ueda HR. Advanced CUBIC protocols for whole-brain and whole-body clearing and imaging. Nat Protoc. 2015;10: 1709–1727.

3. Wang Q, Ding S-L, Li Y, Royall J, Feng D, Lesnar P, et al. The Allen Mouse Brain Common Coordinate Framework: A 3D Reference Atlas. Cell. 2020;181: 936-953.e20.

4. Abraham A, Pedregosa F, Eickenberg M, Gervais P, Mueller A, Kossaifi J, et al. Machine learning for neuroimaging with scikit-learn. Front Neuroinform. 2014;8: 14.

5. Tibshirani R. Regression Shrinkage and Selection via the Lasso. J R Stat Soc Series B Stat Methodol. 1996;58: 267–288.

6. Numa C, Nagai H, Taniguchi M, Nagai M, Shinohara R, Furuyashiki T. Social defeat stress-specific increase in c-Fos expression in the extended amygdala in mice: Involvement of dopamine D1 receptor in the medial prefrontal cortex. Sci Rep. 2019;9: 16670.

Supplementary Figures

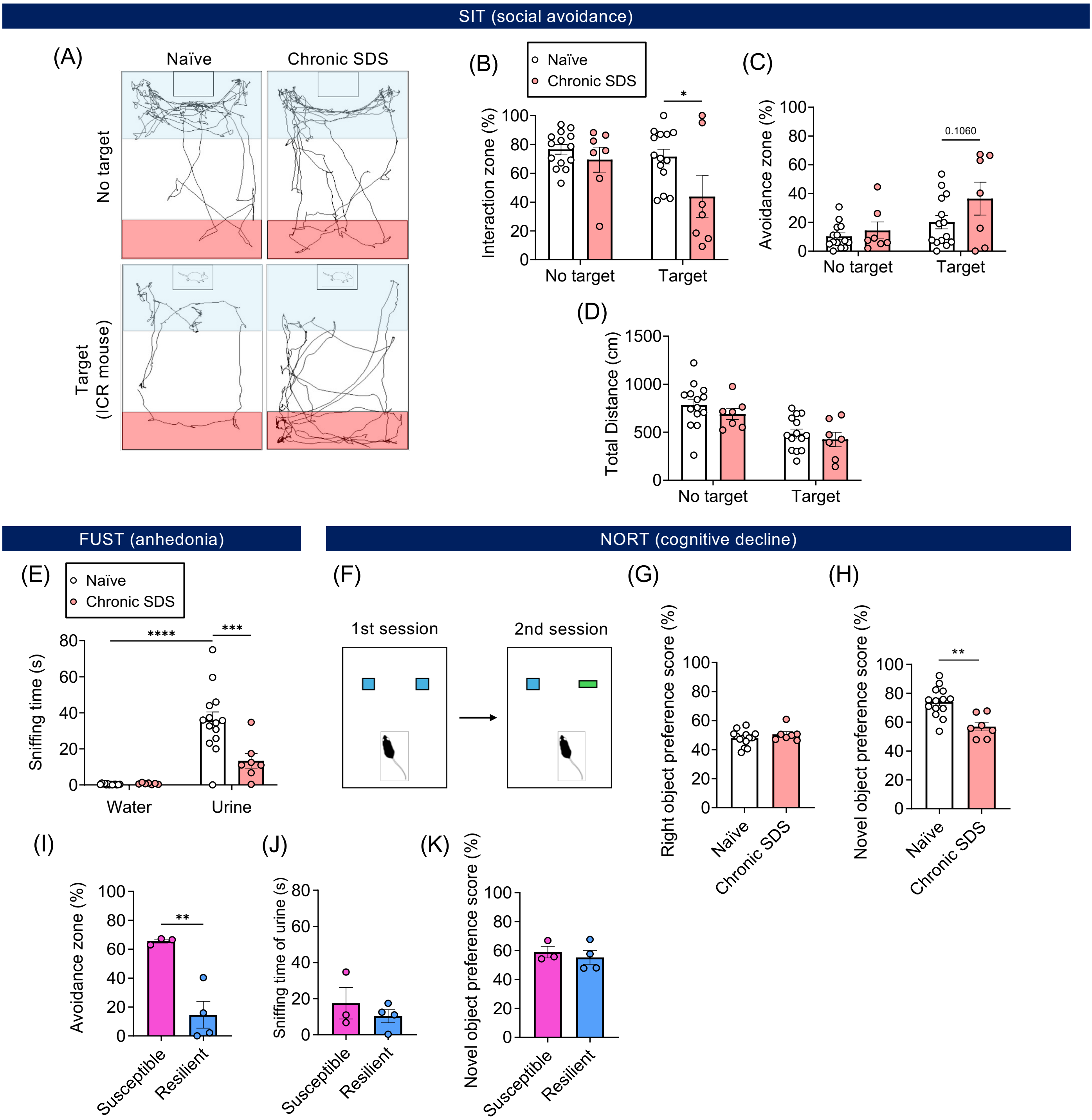

Supplementary Fig. 1. Chronic social defeat stress induces behavioral disturbance.
Naïve and chronic SDS mice were subjected to the social interaction test (SIT; A-D, and I), the female urine sniffing test (FUST; E and J), and the novel object recognition test (NORT; F-H, and K), as shown in Figure 1A. Behavioral indices shown in these graphs are described in the legend of Fig. 3E-I. For the social interaction test, representative traces of ambulation of naïve and chronic SDS mice without or with a target ICR mouse (No target or Target, respectively) are shown in (A). Blue and red rectangles represent the interaction zone and avoidance zone, respectively. For the novel object recognition test, the experimental design is shown in (F). Blue squares and a green rectangle represent Lego blocks and a sand-filled flask, respectively. N = 14 for naïve mice, N = 7 for chronic SDS mice. Chronic SDS mice were categorized into susceptible and resilient mice, and behavioral results of these mice are shown in I-K. N = 3 for Susceptible mice N = 4 for Resilient mice. Two-way repeated measures ANOVA results are shown in Supplementary Table S3. *P < 0.05, ***P < 0.001, ****P < 0.0001 for Bonferroni's multiple comparisons test (B-E). **P < 0.01 for unpaired t-test (H and I). Error bars represent means ± SEM.

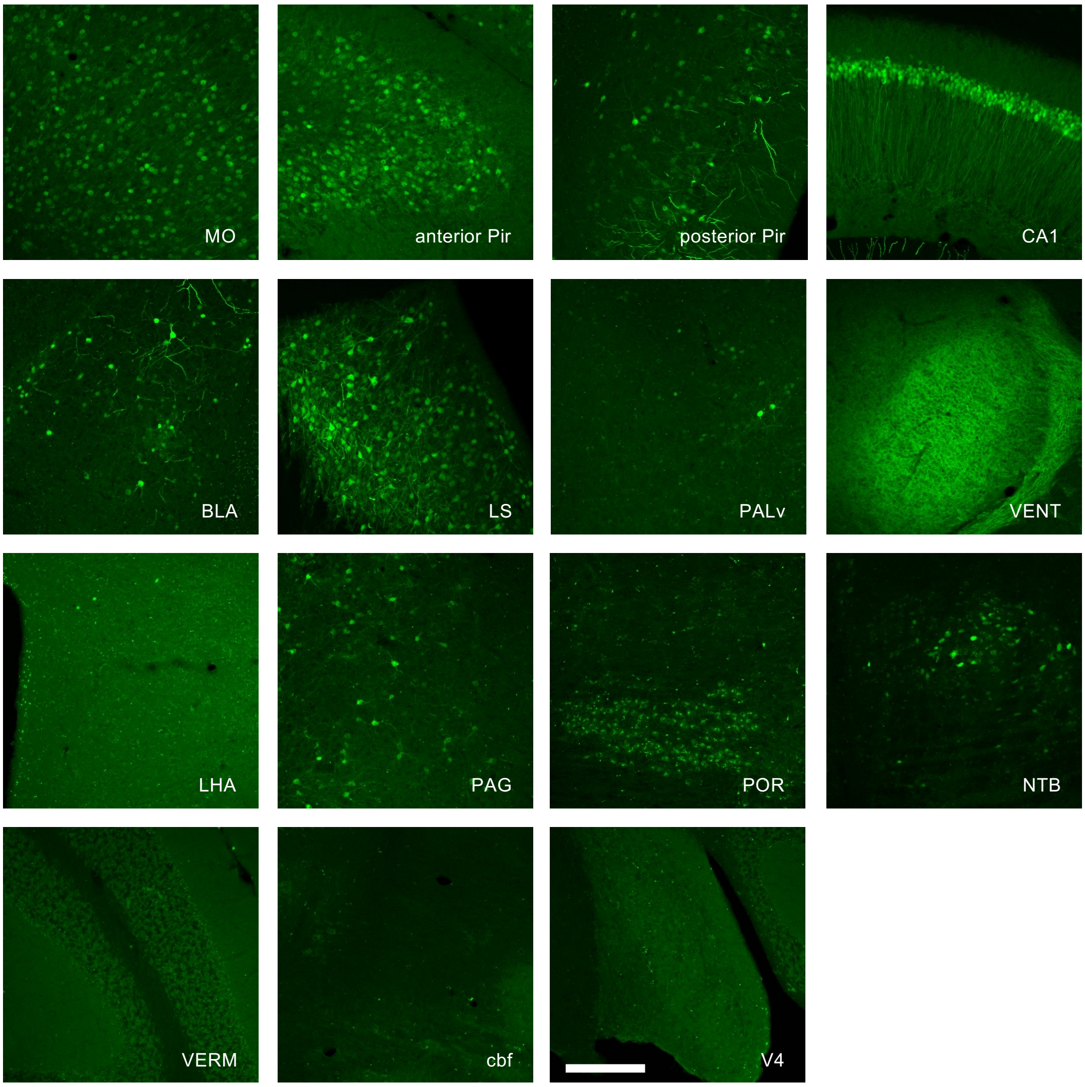

Supplementary Fig. 2. dVenus expression and spatial distribution in various brain regions of Arc-dVenus mice.

Representative images of dVenus signals in various brain regions of Arc-dVenus mice are shown. Abbreviations for brain regions are shown in Supplementary Table 1. Scale bar, 100 μm.

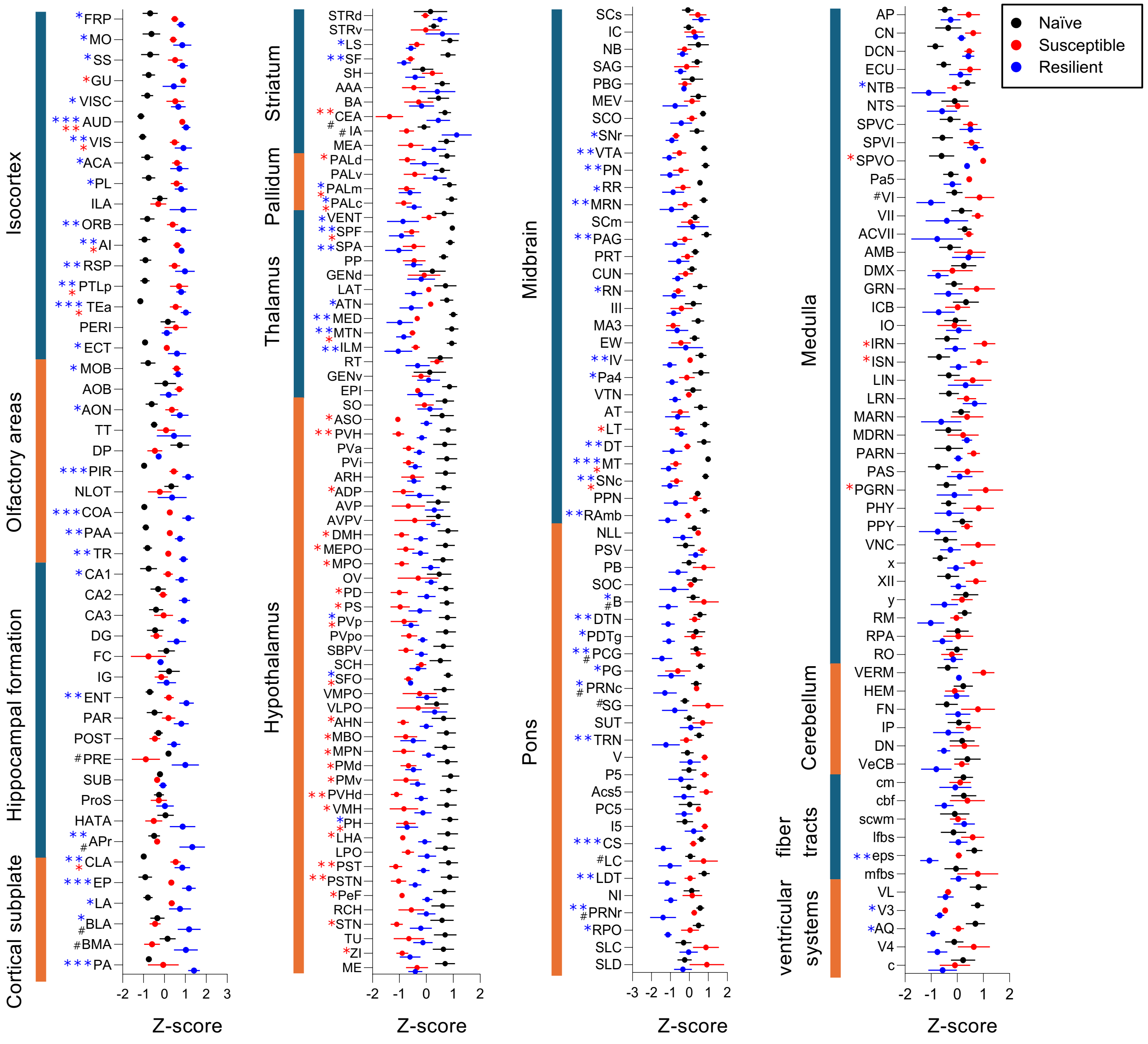

Supplementary Fig. 3. dVenus expression in various brain regions of susceptible and resilient Arc-dVenus mice after social defeat stress.

Z-scores of dVenus fluorescent intensities in the brain of Arc-dVenus mice in Naïve mice (black circle) or in susceptible (red circle) and resilient (blue circle) mice after chronic SDS. Z-scores were the same as shown in Figure 1C, although those of chronic SDS mice were separated to those of susceptible and resilient mice. N=7 for Naïve mice, N=3 for susceptible mice, N=4 for resilient mice. Brain regions were grouped into broader brain structures according to the Allen Mouse Brain Atlas. Abbreviations for brain regions are shown in Supplementary Table S1. Two-way ANOVA results are shown in Supplementary Table S3. $*P < 0.05$, $**P < 0.01$, $***P < 0.001$ for Tukey's multiple comparisons test between Naïve mice and susceptible or resilient mice (red or blue, respectively) and $\#P < 0.05$ for the same test between susceptible and resilient mice. Error bars represent means $\pm$ SEM.

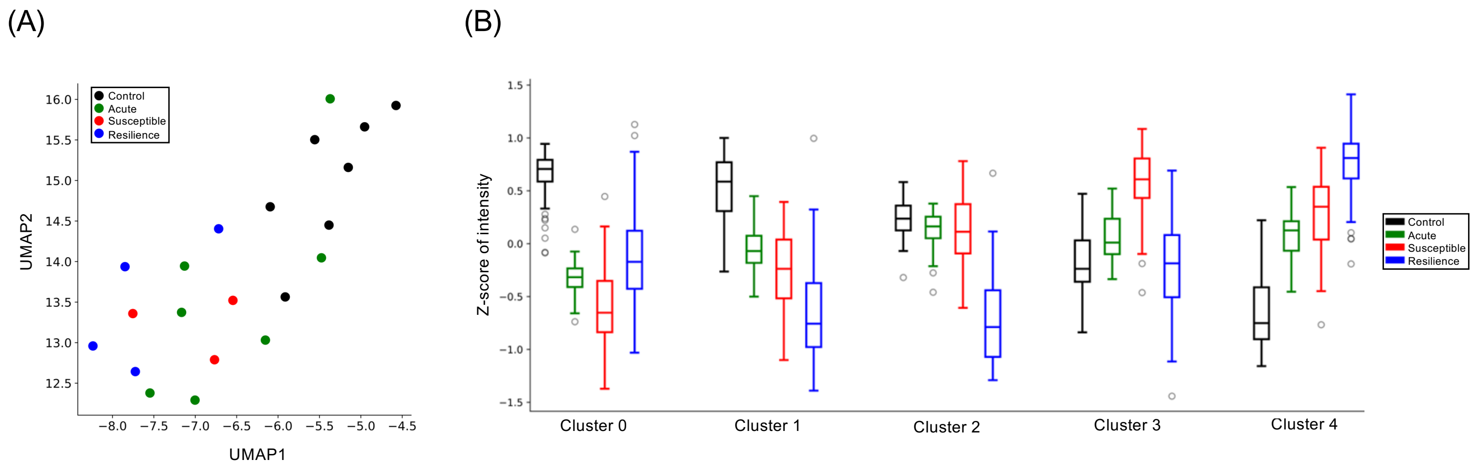

Supplementary Fig. 4. Widely distributed brain regions differently contribute to stress susceptibility after chronic social defeat stress.

(A) UMAP plot to visualize the variability of brain-wide neural activities among Naïve mice (black), acute SDS mice (green), and susceptible (red) and resilient (blue) mice after chronic SDS, based on fluorescent intensities of Arc-dVenus mice. (B) Box plots of z-scores of neural activities in brain regions categorized to the indicated clusters defined in Figure 2C for Naïve mice (black), acute SDS mice (green), and susceptible (red) and resilient (blue) mice after chronic SDS. Z-scores were the same as shown in Figure 2D, although those of chronic SDS mice were separated to those of susceptible and resilient mice. N = 7 for Naïve mice, N=7 for acute SDS mice, N=3 for susceptible mice, N=4 for resilient mice. Refer to the legend of Figure 2 for details.

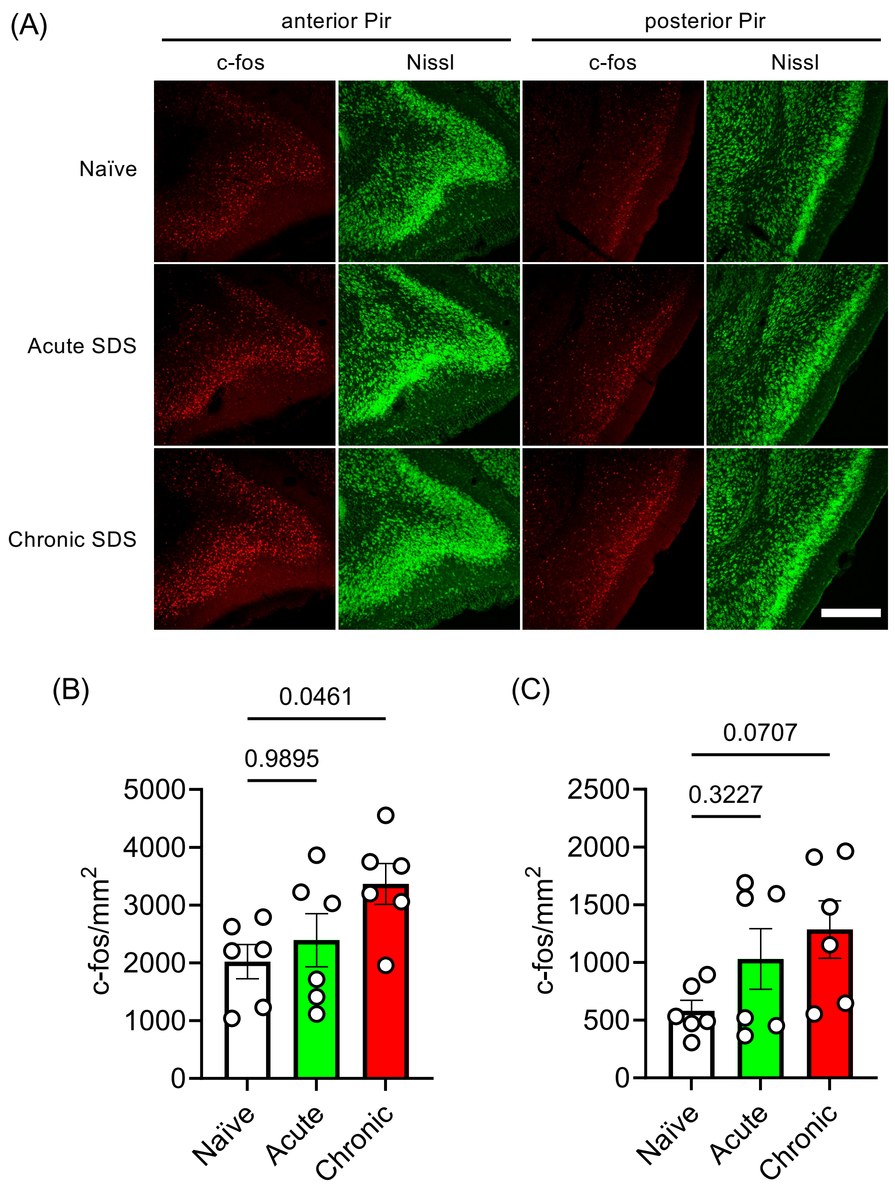

Supplementary Fig. 5. c-Fos expression in the piriform cortex in response to acute and chronic social defeat stress.

(A) Representative images of c-Fos-positive cells (red) with fluorescent Nissl staining (green) in anterior and posterior piriform cortices (Pir) of naïve, acute SDS, and chronic SDS mice. The density of c-Fos-positive cells in these images is shown in Figure 3B. Scale bar, 200 μm. (B, C) The density of c-Fos-positive cells in the anterior (B) and posterior (C) piriform cortices of Naïve, acute SDS, and chronic SDS mice. N = 6 in each group. One-way ANOVA results are shown in Supplementary Table S3. *P* values for Bonferroni's multiple comparisons test. Error bars represent means ± SEM.

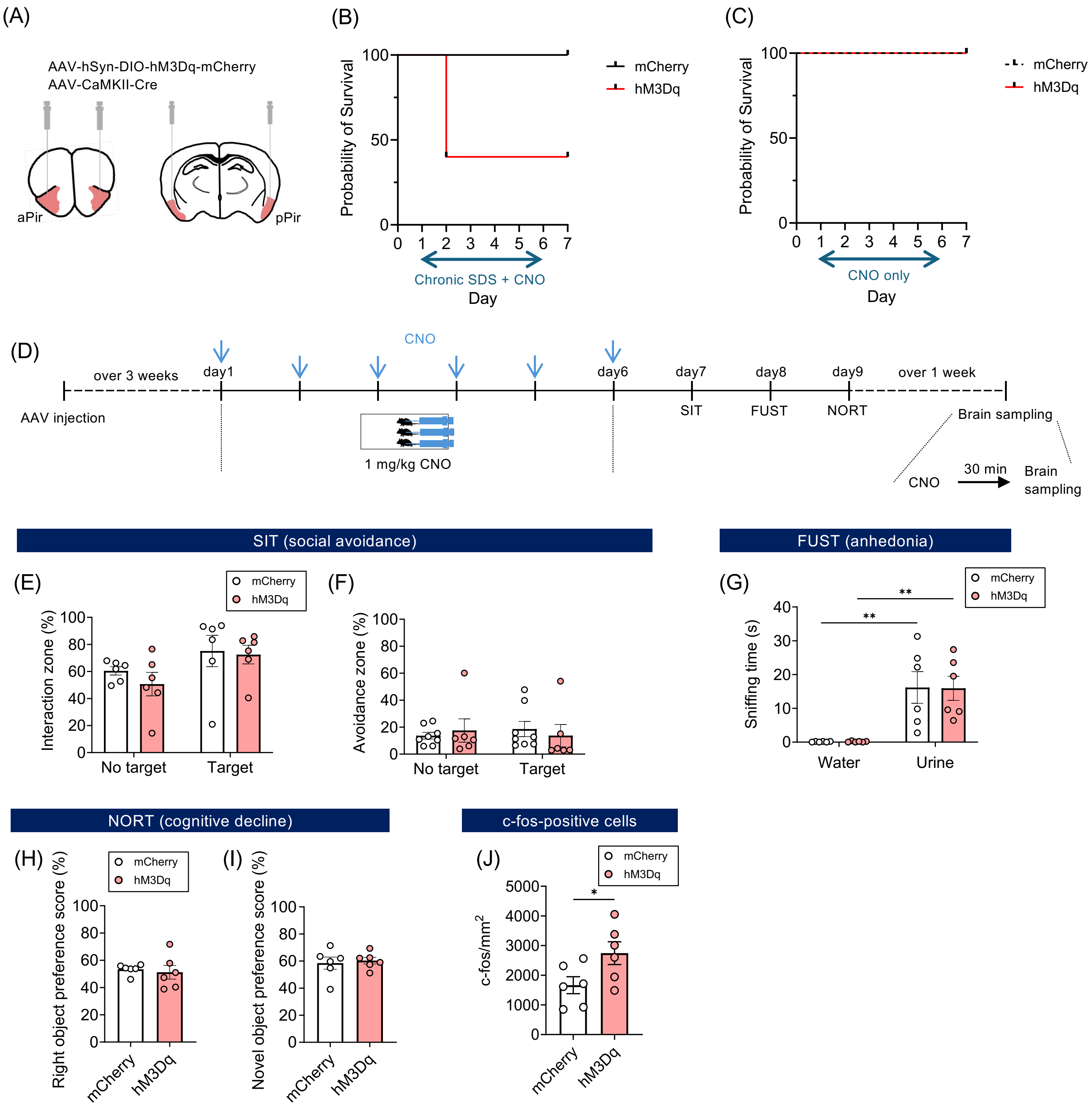
(A)
AAV-hSyn-DIO-hM3Dq-mCherry
AAV-CaMKII-Cre
aPir
pPir
(B)
Probability of Survival
mCherry
hM3Dq
Chronic SDS + CNO
Day
(C)
Probability of Survival
mCherry
hM3Dq
CNO only
Day
(D)
CNO
over 3 weeks
day1
day6
day7
day8
day9
over 1 week
AAV injection
SIT
FUST
NORT
Brain sampling
1 mg/kg CNO
CNO
30 min
Brain sampling
SIT (social avoidance)
FUST (anhedonia)
(E)
Interaction zone (%)
No target
Target
(F)
Avoidance zone (%)
No target
Target
(G)
Sniffing time (s)
Water
Urine
NORT (cognitive decline)
c-fos-positive cells
(H)
Right object preference score (%)
(I)
Novel object preference score (%)
(J)
c-fos/mm2

Supplementary Fig. 6. The activation of the piriform cortex is not sufficient to induce behavioral changes.

(A) AAV vector injection for DREADD-mediated activation of excitatory neurons in the piriform cortex. AAV-hSyn-DIO-hM3Dq-mCherry was stereotaxically injected with AAV-CaMKII-Cre into anterior and posterior piriform cortices (aPir and pPir, respectively). (B, C) Probability of survival of mice without (mCherry) or with (hM3Dq) the DREADD-mediated activation over 7 days with (B) or without (C) chronic SDS. All mice received CNO injections. N = 5 for each group in B, N = 6 for each group in C. (D) Experimental schedule for DREADD-mediated activation of excitatory neurons in the piriform cortex without chronic SDS and subsequent behavioral tests. Three weeks after AAV vector injection, mice received daily CNO administration for 6 days (blue arrows, Day 1 to Day 6). The social interaction test (SIT, Day 7), female urine sniffing test (FUST, Day 8), and novel object recognition test (NORT, Day 9) were conducted without CNO administration. After 1 week or more for recovery, mice received an additional CNO administration before brain sampling for c-Fos immunofluorescent staining. (E-I) Results of the behavioral tests. Mice expressing mCherry alone or hM3Dq-mCherry were subjected to the behavioral tests as shown in (D). Behavioral indices shown in these graphs are described in the legend of Fig. 3E-I. N = 6 in each group. (J) The effect of DREADD-mediated activation on c-Fos expression in the piriform cortex. Mice expressing mCherry alone or hM3Dq-mCherry were subjected to c-Fos immunofluorescent staining. The density of c-Fos-positive cells in the piriform cortex is shown. N = 6 in each group. Two-way repeated measures ANOVA results are shown in Supplementary Table S3. $^{**}P < 0.01$ for Bonferroni's multiple comparisons test (G). $^{*}P < 0.05$ for unpaired t-test (J). Error bars represent means ± SEM.

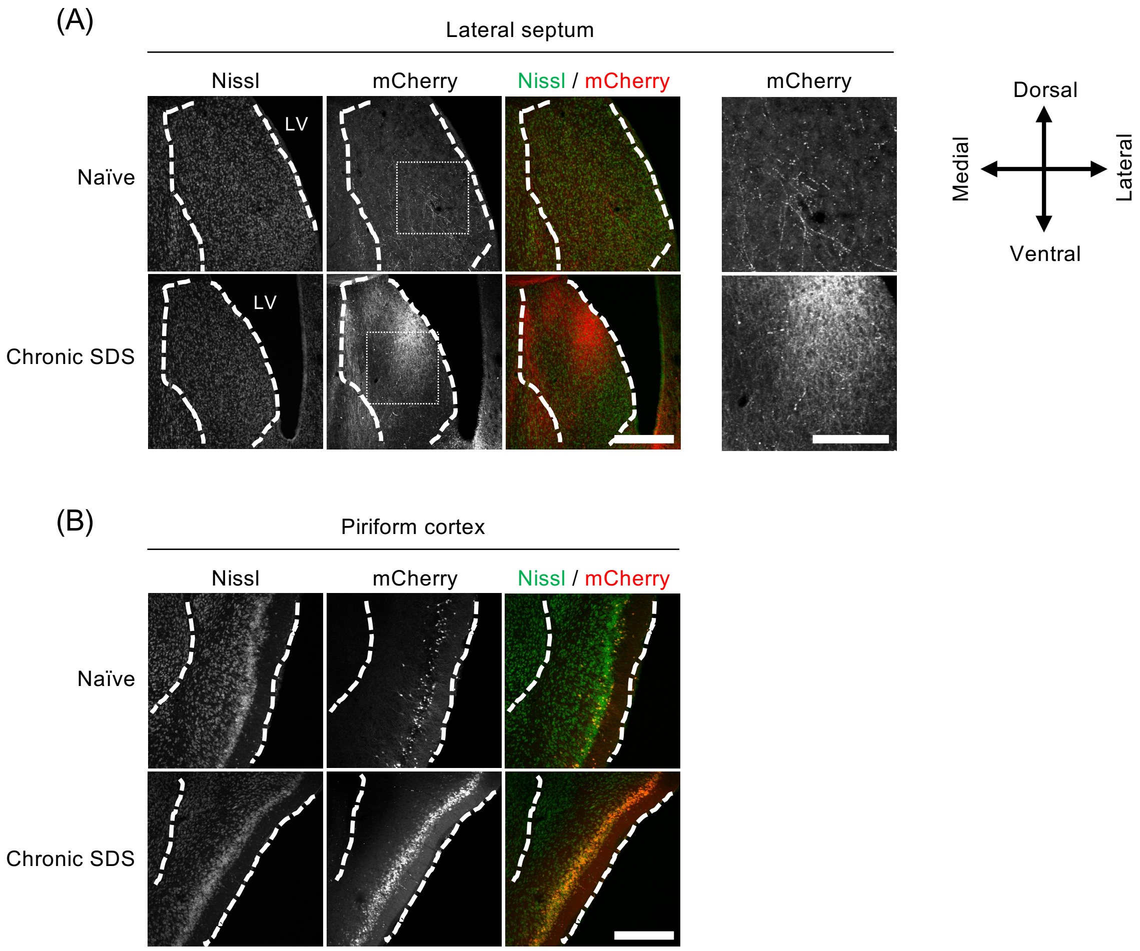

Supplementary Fig. 7. Chronic social defeat stress alters the distribution of mCherry signals derived from axons of the piriform excitatory neurons in the lateral septum.

Representative images show mCherry-positive axons originating from piriform excitatory neurons in the lateral septum (A) and mCherry-positive excitatory neurons in the piriform cortex (B) of Naïve and chronic SDS mice. Mice expressing mCherry alone from Supplementary Fig. 6 (Naïve) and Fig. 4 (chronic SDS) were sacrificed for immunofluorescent staining. Dashed lines outline the lateral septum (A) and the piriform cortex (B), respectively. In the merged images, Nissl and mCherry signals are displayed in green and red, respectively. Scale bars: 200 μm. Enlarged views of the regions within the dotted squares are shown in the rightmost panels in (A). Scale bar, 100 μm. LV: lateral ventricle.

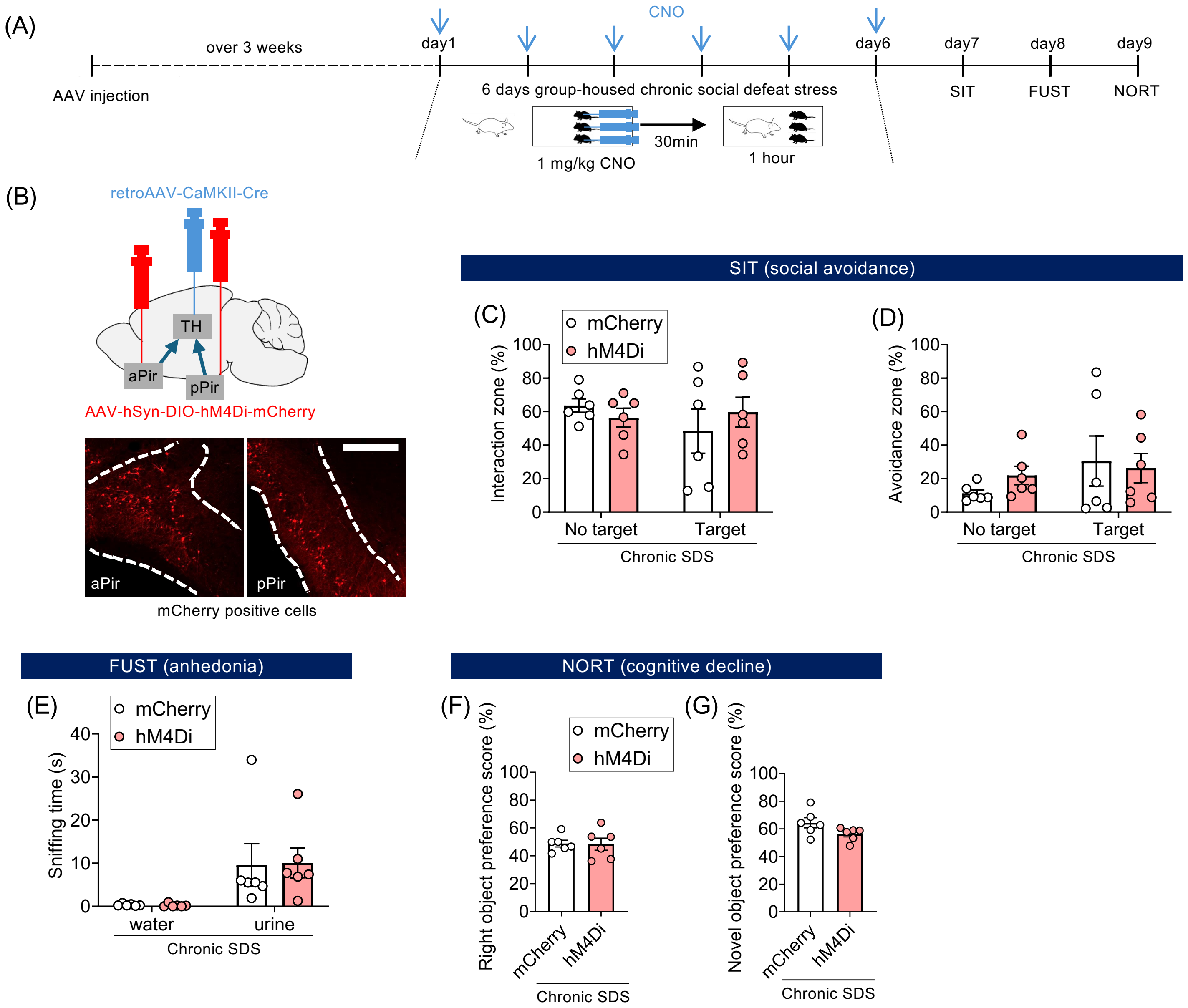

Supplementary Fig. 8. The inhibition of the piriform cortex to non-specific thalamic nuclei pathway during chronic social defeat stress has no obvious effect on behaviors.

(A) Experimental schedule for DREADD-mediated inhibition of the piriform cortex to non-specific thalamic nuclei pathway during chronic SDS and subsequent behavioral tests. The experiments were scheduled as described in the legend of Figure 3C. (B) AAV vector injection for DREADD-mediated inhibition of excitatory neurons in the piriform cortex projecting to non-specific thalamic nuclei. AAV-hSyn-DIO-hM4Di-mCherry and retroAAV-CaMKII-Cre were stereotaxically injected into anterior and posterior piriform cortices (aPir and pPir, respectively) and non-specific thalamic nuclei (TH), respectively (upper panel). Representative images of mCherry-positive cells in the aPir and pPir are shown (lower panels). Scale bar, 200 µm. (C-G) Results of the behavioral tests. Mice expressing mCherry alone or hM4Di-mCherry in the targeted neurons and administered with CNO during chronic SDS (mCherry+CNO or hM4Di+CNO, respectively) were subjected to the behavioral tests as shown in (A). Behavioral indices shown in these graphs are described in the legend of Fig. 3E-I. Two-way repeated measures ANOVA results are shown in Supplementary Table S3. N = 6 in each group. Error bars represent means ± SEM.

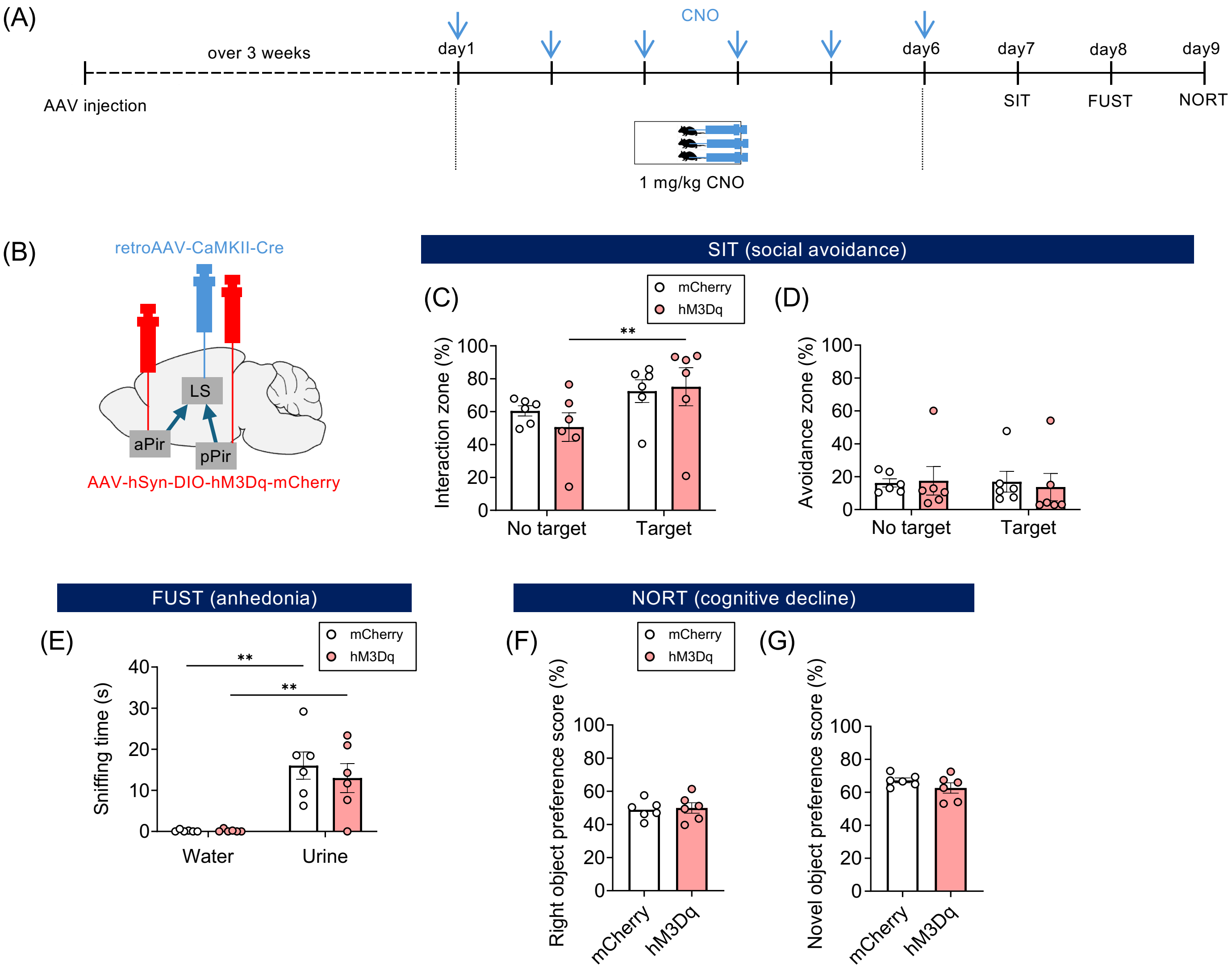

Supplementary Fig. 9. The activation of the piriform cortex to lateral septum pathway without social defeat stress is not sufficient to induce behavioral changes.

(A) Experimental schedule for DREADD-mediated activation of the piriform cortex to lateral septum pathway and subsequent behavioral tests without SDS. The experiments were scheduled as described in the legend of Supplementary Figure 6D. (B) AAV vector injection for DREADD-mediated activation of excitatory neurons in the piriform cortex projecting to the lateral septum. AAV-hSyn-DIO-hM3Dq-mCherry and retroAAV-CaMKII-Cre were stereotaxically injected into anterior and posterior piriform cortices (aPir and pPir, respectively) and the lateral septum (LS), respectively. (C-G) Results of the behavioral tests. Mice expressing mCherry alone or hM3Dq-mCherry were subjected to the behavioral tests as shown in (A). Behavioral indices shown in these graphs are described in the legend of Fig. 3E-I. N = 6 in each group. Two-way repeated measures ANOVA results are shown in Supplementary Table S3. $^{**}P < 0.01$, for Bonferroni's multiple comparisons test. Error bars represent means ± SEM.

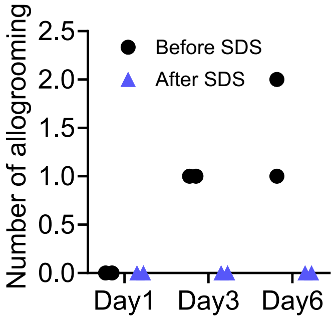

Supplementary Fig. 10. Social defeat stress increases allogrooming in the home cage. Allogrooming was counted over a one-hour period in each home cage containing three defeated mice, both before (black circles) and after (blue triangles) each SDS exposure on Days 1, 3, and 6. The total number of allogrooming in each cage was determined for two cages, with individual dots representing data points from the respective cages.

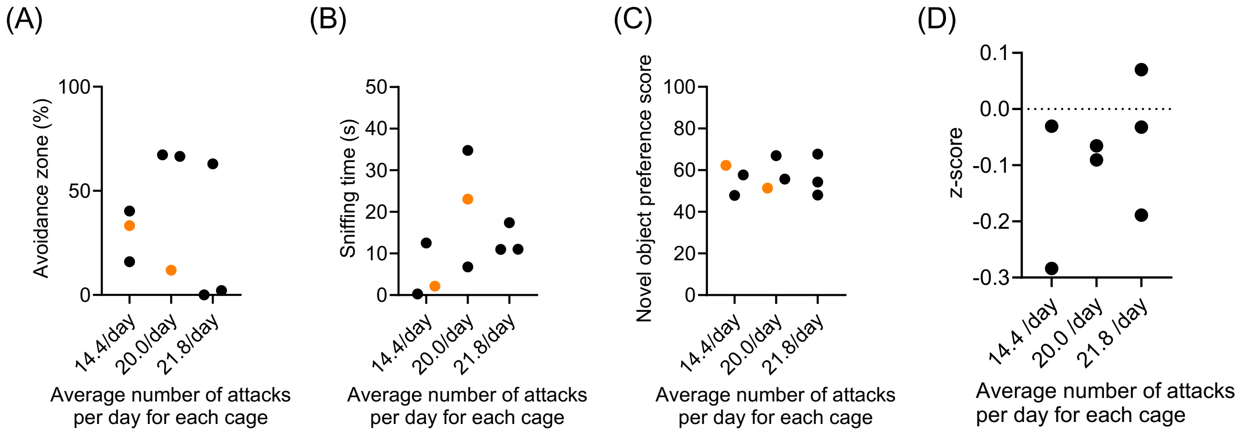

Supplementary Fig. 11. The number of attacks does not correlate with chronic SDS-induced changes in behaviors or Arc-dVenus signals.

(A-C) Relationships between the average number of daily attacks per cage and social avoidance in the social interaction test (A), urine sniffing time in the female urine sniffing test (B), or novel object recognition score in the novel object recognition test (C). The values of Arc-dVenus mice (black circles) and their wild-type littermates (orange circles) are shown. (D) Relationships between the average number of daily attacks per cage and the overall chronic SDS-induced changes in Arc-dVenus signals.
